# Reaction of Perrhenate with Phthalocyanine Derivatives in the Presence of Reducing Agents and Rhenium Oxide Nanoparticles in Biomedical Applications

**DOI:** 10.1002/open.202200037

**Published:** 2022-07-20

**Authors:** Songeziwe Ntsimango, Sendibitiyosi Gandidzanwa, Sinelizwi V. Joseph, Eric C. Hosten, Marvin Randall, Adrienne L. Edkins, Samson M. Khene, Philani Mashazi, Tebello Nyokong, Abubak'r Abrahams, Zenixole R. Tshentu

**Affiliations:** ^1^ Department of Chemistry Nelson Mandela University Gqeberha 6001 South Africa; ^2^ Chemistry and Molecular Sciences Institute School of Chemistry University of Witwatersrand Johannesburg 2050 South Africa; ^3^ Electron Microscopy Unit Rhodes University Makhanda 6140 South Africa; ^4^ Biomedical biotechnology Research Unit (BioBRU), Department of Biochemistry and Microbiology Rhodes University Makhanda 6140 South Africa; ^5^ Department of Chemistry Rhodes University Makhanda 6140 South Africa; ^6^ Institute for Nanotechnology Innovation Rhodes University Makhanda 6140 South Africa

**Keywords:** hydrolytic cleavage, metalation, phthalocyanines, rhenium, rhenium oxide nanoparticles

## Abstract

A novel alternative route to access rhenium(V)−phthalocyanine complexes through direct metalation of metal‐free phthalocyanines (H_2_Pcs) with a rhenium(VII) salt in the presence of various two‐electron reducing agents is presented. Direct ion metalation of tetraamino‐ or tetranitrophthalocyanine with perrhenate (ReO_4_^−^) in the presence of triphenylphosphine led to oxidative decomposition of the H_2_Pcs, giving their respective phthalonitriles. Conversely, treatment of H_2_Pcs with ReO_4_^−^ employing sodium metabisulfite yielded the desired Re^V^O−Pc complex. Finally, reaction of H_2_Pcs with ReO_4_^−^ and NaBH_4_ as reducing agent led to the formation of rhenium oxide (Re_x_O_y_) nanoparticles (NPs). The NP synthesis was optimised, and the Re_x_O_y_ NPs were capped with folic acid (FA) conjugated with tetraaminophthalocyanine (TAPc) to enhance their cancer cell targeting ability. The cytotoxicity profile of the resultant Re_x_O_y_−TAPc−FA NPs was assessed and found to be greater than 80 % viability in four cell lines, namely, MDA−MB‐231, HCC7, HCC1806 and HEK293T. Non‐cytotoxic concentrations were determined and employed in cancer cell localization studies. The particle size effect on localization of NPs was also investigated using confocal fluorescence and transmission electron microscopy. The smaller NPs (≈10 nm) were found to exhibit stronger fluorescence properties than the ≈50 nm NPs and exhibited better cell localization ability than the ≈50 nm NPs.

## Graphical abstract

**The reaction of phthalocyanine derivatives with perrhenate** in the presence of triphenylphosphine yields phthalonitriles. Tetraaminophthalocyanine was directly metalated with rhenium(IV) via reduction of perrhenate with sodium metabisulfite to form a rhenium(V) phthalocyanine complex. The folate−tetraaminophthalocyanine conjugate was used to functionalize Re_x_O_y_ nanoparticles formed from the reduction of perrhenate by sodium borohydride. The Re_x_O_y_ nanoparticles were employed in folate receptor cancer cell internalization.

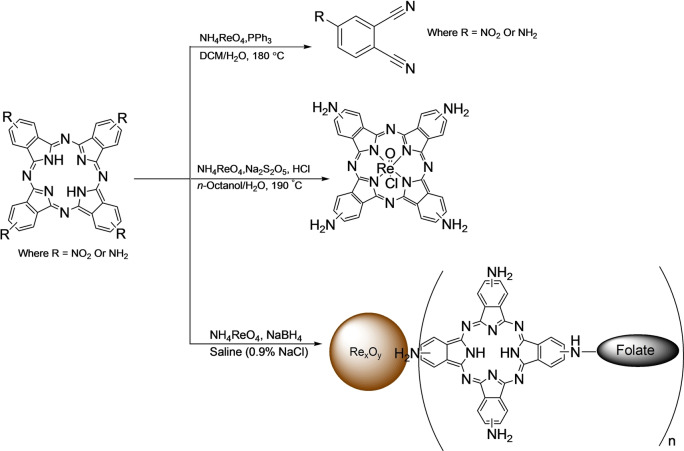

## Introduction

Different metals have been successfully incorporated into the phthalocyanine cavity yielding metallophthalocyanines (MPcs). Owing to their versatility and interesting material properties, metallophthalocyanines have made their way to the medical industry.^[1]^ Conventionally, MPcs are extensively employed as photosensitizers,^[2]^ in dye‐sensitized solar cells,^[3]^ and as photocatalysts,^[4]^ among a myriad of other applications.^[5]^ Despite the wide study of MPcs, literature on rhenium phthalocyanines is scarce and limited to oxo, halogeno, and nitrido complexes.^[6]^ Generally, MPcs retain a *D*_4*h*_ symmetry owing to their planar geometry. However, the rhenium phthalocyanines (RePcs) possess a *C*_4*v*_ symmetry due to the larger size of the Re^V^ ion, which does not fit in the Pc cavity, making RePcs possess an electric dipole that is perpendicular to the Pc plane,^[7]^ resulting in the incorporation of two‐valent Re and dimerization [PcRe≡RePc].^[8]^ Ziener et al. have also reported on the synthesis of the dimeric RePcs of the type [PcRe−O−RePc], a *μ*‐oxo dimetallate complex from rhenium pentachloride and 4‐*tert*‐butylphthalodinitrile.^[9]^ Monomeric RePc has also been reported in the literature. For example, Ziener et al. developed a synthesis of unsubstituted RePc from NH_4_ReO_4_ and phthalodinitrile to form PcReN^[9]^ and substituted (*t*‐Bu)_4_PcReO(OEt) complex from the condensation of 4‐*tert*‐butylphthalodinitrile in the presence of NH_4_ReO_4_ and a suitable reducing agent.^[9]^

The most recent synthesis of monomeric RePcs was achieved with the amido group attached to the metal centre and occupying the axial position, with the macrocycle in the equatorial position.^[6]^ All the above‐mentioned examples involve the synthesis of rhenium−phthalocyanines through tetracyclization of phthalocyanine precursors with rhenium salts. None of them involve the direction metalation of H_2_Pcs with rhenium metal ions to form rhenium−phthalocyanine complexes and very few oxidorhenium(V)−Pc complexes have been isolated.^[9]^ In this paper, we report on our effort to directly synthesise Re^V^−Pc complexes via the direct metalation of H_2_Pcs, starting from perrhenate in the presence of various reducing agents. To this end, different results were obtained, including the formation of rhenium oxide (Re_x_O_y_) nanoparticles upon reduction of NH_4_ReO_4_ with NaBH_4_.

Lower oxidation state rhenium oxides were previously observed by Broadbent and Johnson from a reaction of perrhenate and sodium borohydride.^[10]^ In this study, we attempted to form oxidorhenium(V)−Pc complexes by reduction of perrhenate with sodium borohydride in the presence of free Pcs and the rhenium oxide nanoparticles were isolated with rhenium in different oxidation states. Given the possible radiopharmaceutical application of Re‐186/188,^[11]^ we explored their potential as nanomedicine drugs by studying their localization in cancer cells for targeted nanoradiopharmaceutical applications. Generally, nanoparticles possess the ability to overcome the shortcomings that are presented by traditional drugs such as drug resistance at the cellular level, distribution, clearance of anticancer drugs in the body, and drug resistance at the tumour level due to physiological barriers and biotransformation.^[12]^ Chelated compounds of Re‐186/188 have been used for bone palliation, intravascular radiation therapy, and treatment of medullar carcinoma, and this highlights the prospects of Re‐186/188 in medical applications.[^13^, ^14^] Rhenium stands as a good candidate for nanoradiopharmaceutical development because of its good nuclear properties, physical half‐life, and decay characteristics.^[15]^

The interactions of nanoparticles with biological systems like living cells have encouraged a collaborative research across various disciplines such as chemistry, physics, materials science and biology.^[15]^ Various studies illustrating nanoparticle behaviour in biological systems have been conducted using confocal fluorescence microscopy and other imaging techniques such as transmission electron microscopy (TEM). For example, Gliga et al. investigated the cell‐uptake of silver nanoparticles and their intracellular localization in human lung cells using TEM.^[16]^ Jin et al. employed confocal fluorescence microscopy to investigate the specific uptake of epidermal growth factor, EGF‐ conjugated nanoparticles in lung cancer cells.^[17]^ Despite the abundant literature on cell localization studies of various nanoparticle systems, there is a lack of information on the cell‐uptake of rhenium oxide nanoparticles and of cell accumulation studies. Herein, folate‐functionalized rhenium oxide nanoparticles were synthesized and shown to be taken up in cancer cells. We also report on the synthesis of an oxidorhenium(V)−phthalocyanine complex via direct reductive metalation of metal‐free phthalocyanine with rhenium in its perrhenate (ReO_4_^−^) form as well as the rhenium(V)‐mediated hydrolysis of the Pcs to form phthalonitriles which can be a useful strategy for the recovery of the precursor compounds

## Results and Discussion

### Synthesis and Characterisation of TAPc and TAPc−FA

Phthalocyanines were prepared by tetracyclomerization of 4‐nitrophthalonitrile around zinc(II) to form TNZnPc. The reduction of nitro substituents on TNZnPc was carried out to form TAZnPc followed by the extrication of the central zinc ion to form metal‐free Pc (TAPc). The success of the synthesis was confirmed by UV‐Vis absorption spectral analysis. The bands for TNZnPc appear at 350 nm and at 700 nm (Figure S1A). Usually, the Q band (700 nm) of metalated Pcs is sharp but the bands are broad for both metalated and metal‐free Pc derivatives due to aggregation. The spectrum of the nitro‐containing zinc phthalocyanine showed a slight splitting of the Q band and this could be due to the strong electron‐withdrawing nitro group. After reduction, a bathochromic shift occurred to 740 nm due to the effect of NH_2_, and an extra band appeared at 450 nm due to charge transfer within the TAZnPc molecules.^[18]^ After dislodging zinc from TAZnPc, TAPc was attained, and the UV‐Vis spectrum showed a very broad brand in the range of 600–800 nm due to aggregation.

The structure of TNZnPc was confirmed further by FTIR spectroscopy (Figure S1B). The FTIR spectrum of TNZnPc has two peaks at 3309 and 3080 cm^−1^, corresponding to the aromatic C−H stretching mode of the phthalocyanine. The peak at 1638 cm^−1^ can be attributed to the C=C macrocycle ring deformation, whereas the peaks at 1531 and 1333 cm^−1^ were ascribed to the nitro groups. The peak at 1615 cm^−1^ is due to the bending of the N−H groups, and the series of peaks seen at 1148, 1109, 1069 cm^−1^ belong to the C−H bending (in‐plane deformation), isoindole C−N stretching and C−N in‐plane bending, respectively. Upon the reduction of TNZnPc to TAZnPc, peaks at 3300, 3290 and 1620 cm^−1^ were observed, which correspond to the stretching vibrations of the amino N−H groups. The disappearance of the peaks at 1531 and 1333 cm^−1^ attributed to the nitro groups further confirmed the reduction of nitro groups to amino groups. A similar FTIR spectrum was obtained for a metal‐free TAPc, however, with additional NH bending vibrations of the indole groups.

**Scheme 1:**
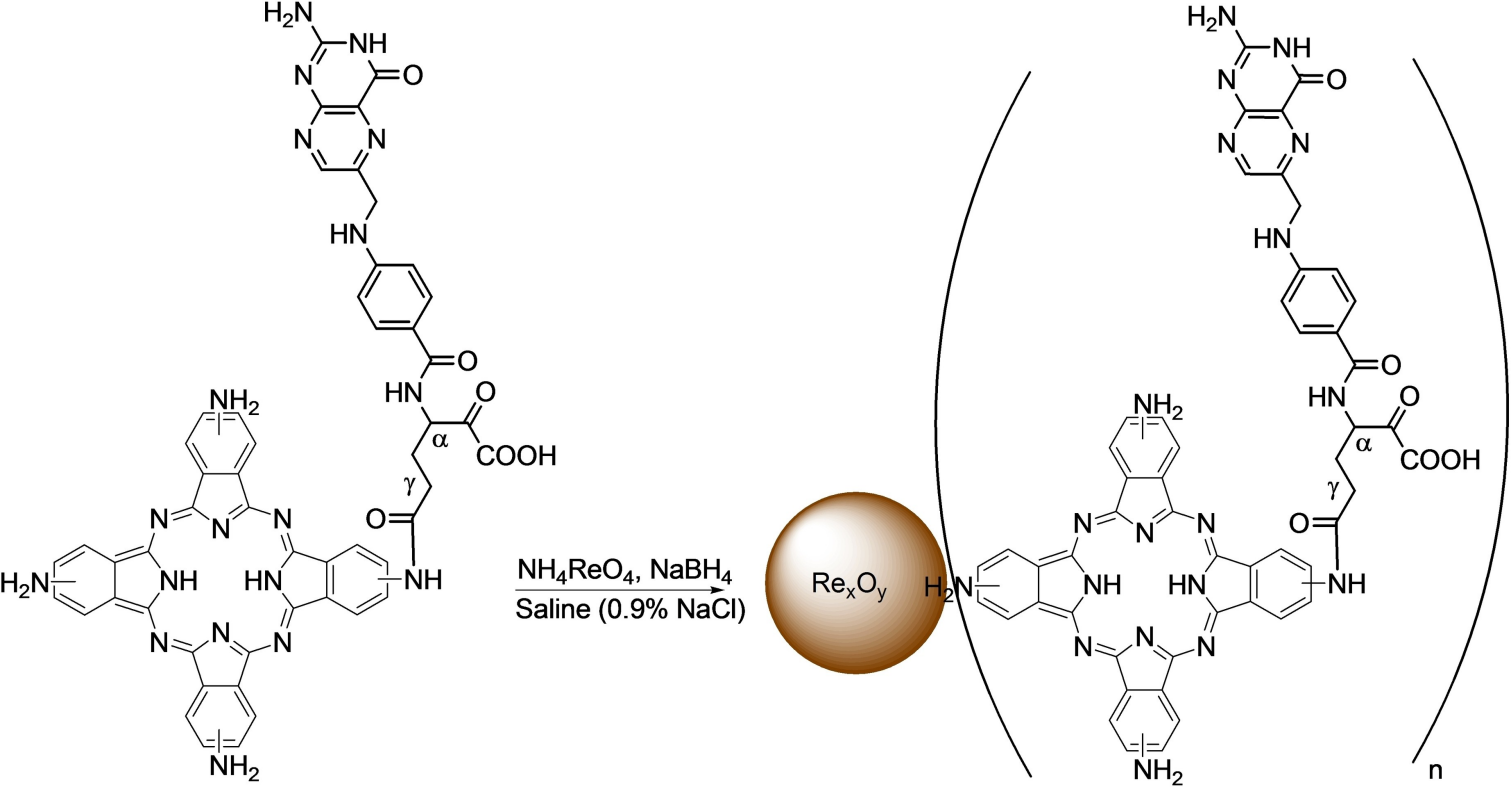
Synthesis of folate‐conjugated tetraaminophthalocyanine‐capped (TAPc−FA) rhenium oxide nanoparticles (Re_x_O_y_ NPs) using ammonium perrhenate as the precursor and sodium borohydride as the reducing agent. The α‐conjugates are also formed and presented in Scheme S3 (Supporting Information).

**Figure 1:**
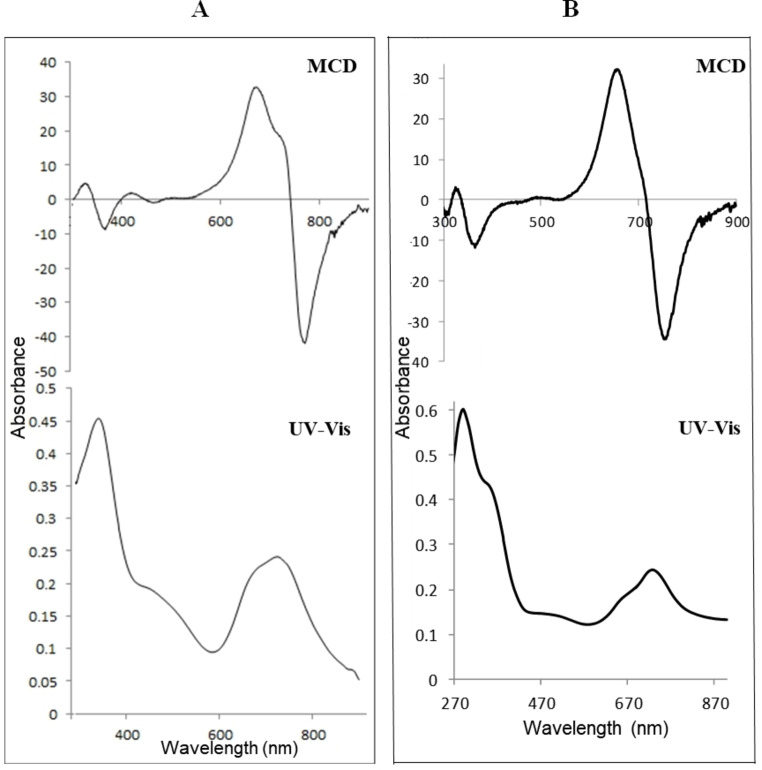
UV‐Vis and MCD spectra of (A) TAPc and (B) TAPc−FA.

The conjugation of TAPc with folate was achieved by the activation of the carboxyl group of the folic acid (FA) with *N,N′*‐dicyclohexylcarbodiimide (DCC) (Scheme S2). Folic acid contains two COOH groups, in α and γ positions (Scheme 1), which react with an amine group via amide bond formation. Due to the higher reactivity of the γ‐carboxylic acid group compared to the α‐carboxylic acid, the former is more likely to be activated by DCC.^[19]^ For porphyrin−FA conjugates, it has also been shown that the amide bond mainly forms from the γ‐carboxylic acid. Therefore, in this study it is assumed that the conjugation is dominantly achieved through reaction of the γ‐carboxylic acid. The UV‐Vis spectrum of the FA conjugated TAPc (TAPc−FA) is compared with the UV‐Vis spectrum of TAPc (Figure 1), and there are no significant differences. The Q band of FA−TAPc is slightly red shifted (740 nm) and the B bands are absorbing almost at the same wavelength, that is, 360 nm and 358 nm for TAPc and TAPc−FA, respectively. The spectrum of TAPc−FA also shows a band at 298 nm which could correspond to the π‐transitions of the cyclic part of the folic acid.^[19]^ The magnetic circular dichroism (MCD) electronic spectrum of TAPc−FA is also similar to the TAPc spectrum. Two very broad Faraday A_1_ bands that possess opposite charges appear at 664 and 763 nm. The B_0_ term is also within the range of 300–400 nm as in the case of TAPc, suggesting that the conjugation with FA does not alter the electronic properties of the Pcs to a larger extent (Figure 1).

**Figure 2:**
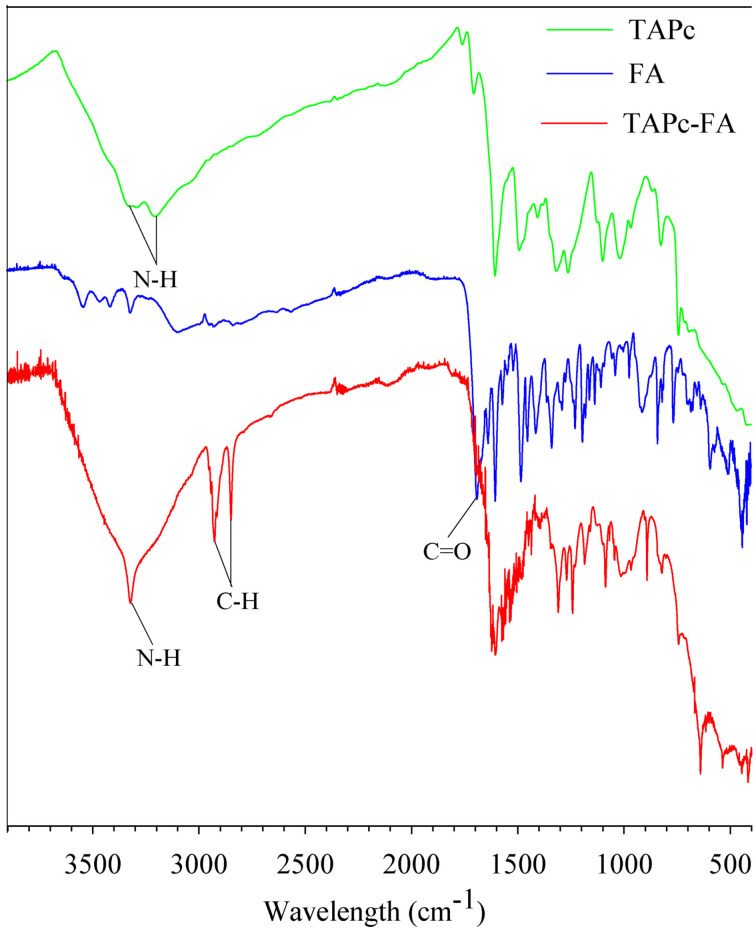
Stacked FTIR spectra of tetraaminophthalocyanine (TAPc), folate (FA) and folate‐conjugated tetraaminophthalocyanine (TAPc−FA).

The FTIR spectra (Figure 2) of TAPc−FA exhibits peaks at 3336 cm^−1^ corresponding to the N−H stretch of the tetraamine substituents, peaks at 2935 and 2853 cm^−1^ that can be attributed to aromatic C−H stretching vibrations, and at 1694 cm^−1^, possibly attributable to the C=O stretching vibration of the amide group. The use of FTIR to confirm TAPc−FA is complicated owing to the amide bonds that are already present on FA; for this reason FTIR cannot be used to conclusively prove the amide bond formation in the case of TAPc.

**Figure 3:**
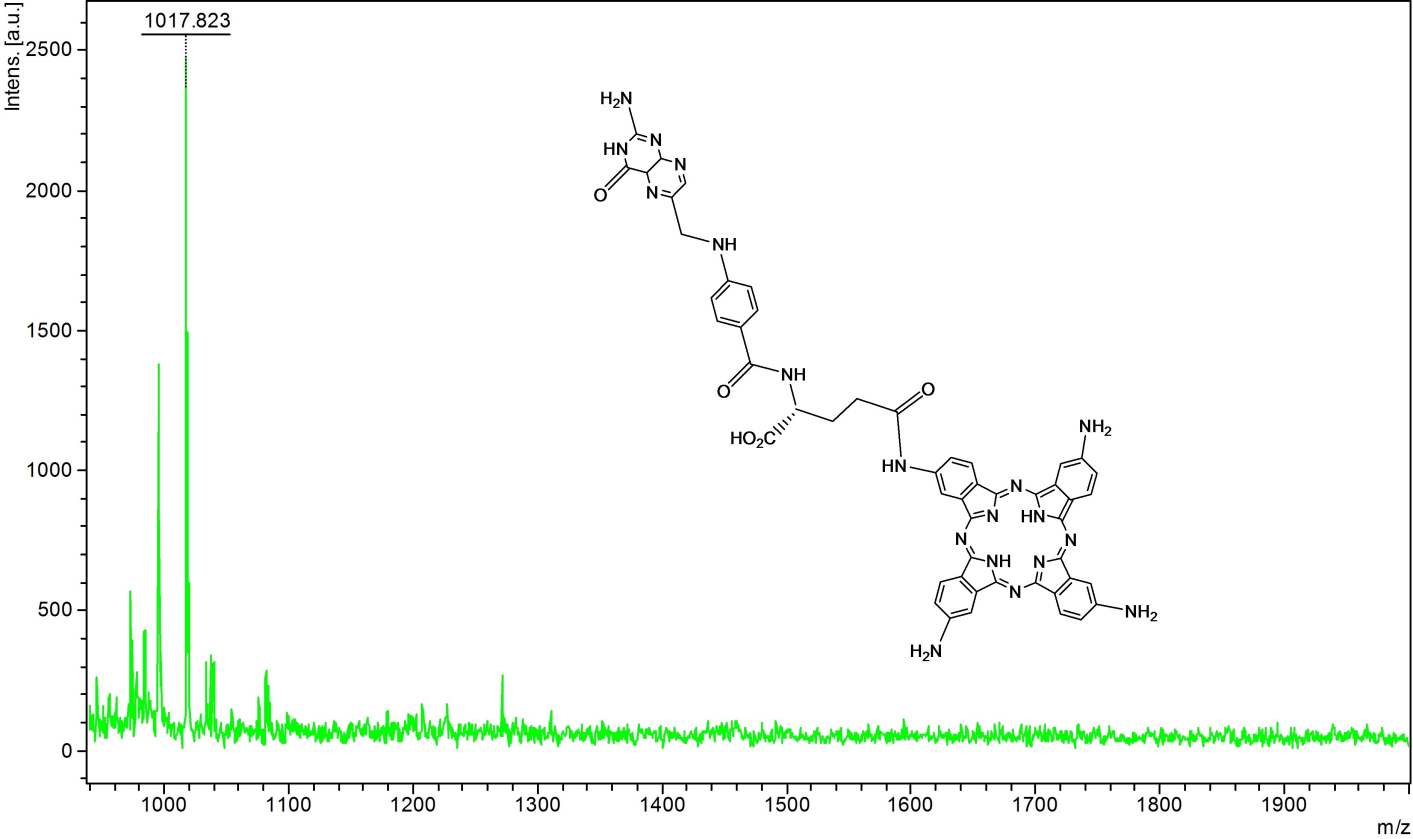
Mass spectrum of folate‐conjugated tetraaminophthalocyanine (TAPc−FA) for the obtained [M+2H]^+^.

The mass spectrum of TAPc−FA (Figure 3) shows a molecular ion at 1017 amu [M+2H]^+^ in agreement with a 1 : 1 Pc:FA ratio. This confirmed the amidation of one of the amine groups in TAPc.

### Rhenium(V)‐Mediated Hydrolysis of Pcs and Characterization of the Phthalonitriles

In an attempt to synthesize rhenium(V)−phthalocyanine complexes through direct metalation of metal‐free phthalocyanine with rhenium in its perrhenate form, using triphenylphosphine as a reducing agent, hydrolysis of a Pc to a phthalonitrile was observed. Pcs are known to have high thermal and chemical stability; however, their decomposition under various conditions has been reported. Linstead et al. reported the decomposition of FePcs into phthalimide in the presence of hot nitric acid or aqua regia.^[20]^ The same result was obtained when cold acidic permanganate was used.^[20]^ Hot concentrated H_2_SO_4_ generated both phthalic acid and phthalimide as the hydrolysis products.^[20]^ A CoPc has also been reported to undergo photochemical decomposition, and it was discovered that the C−N bonds of the Pc cleave at early stages hence leading to the dissociation of the Pc.^[21]^ The conversion of the Pcs to phthalonitriles in this study was confirmed using FTIR (Figure S2A). The existence of the carbonitrile group in the hydrolysis products is evident from the obtained FTIR spectra. For the hydrolysis product of TNPc, a peak was observed at 2240 cm^−1^. A similar peak (at 2255 cm^−1^) was observed in the hydrolysis product of TAPc (Figures S2B and S3). These peaks were attributed to the nitrile group (C≡N).

**Figure 4:**
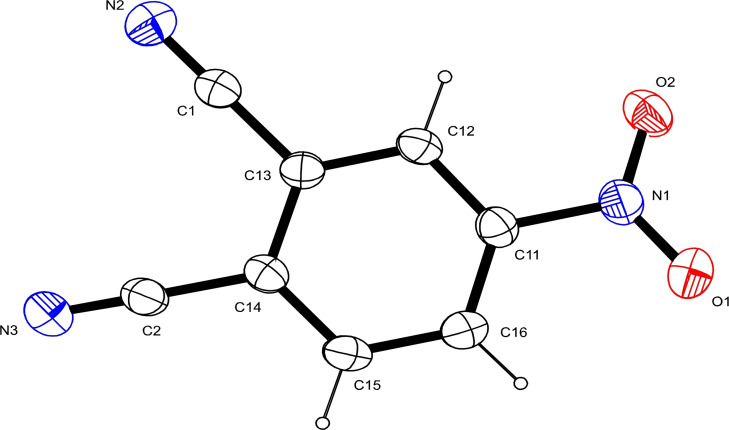
An ORTEP view of the major product of degradation of TNPc (4‐nitrophthalonitrile).

An ORTEP plot of 4‐nitrophthalonitrile (Figure 4) showed bond distances between C−C, from C11 to C16, in the range 1.38(9) to 1.40(1) Å, which correspond well to the average bond length of aromatic C−C bonds. The bond lengths C1−C13 and C2−C14, which were 1.4393(14) and 1.4423(14) Å, respectively, are characteristic of C−C single bonds. The short bond distances between C1−N2 (1.1394(15) Å) and C2−N3 (1.1429(14) Å) suggest C≡N triple bonds as expected for nitriles. Some of the bond distances and bond angles (Table 1) and the metrical parameters of this compound are similar to those published.^[21]^

**Table 1:** Selected bond lengths (Å) and bond angles (°) for 4‐nitrophthalonitrile.

Bond lengths	[Å]	Bond angles	[°]
O(1)−N(1)	1.2206(13)	O(1)−N(1)−O(2)	124.58(10)
O(2)−N(1)	1.2222(12)	O(1)−N(1)−C(11)	117.40(9)
N(1)−C(11)	1.4736(13)	O(2)−N(1)−C(11)	118.02(9)
N(2)−C(1)	1.1394(15)	N(2)−C(1)−C(13)	178.18(12)
N(3)−C(2)	1.1429(14)	N(3)−C(2)−C(14)	178.52(11)
C(1)−C(13)	1.4393(14)	N(1)−C(11)−C(12)	118.06(8)
C(2)−C(14)	1.4423(14)	N(1)−C(11)−C(16)	118.53(9)
C(11)−C(12)	1.3791(13)	C(12)−C(11)−C(16)	123.41(9)

To further confirm the results of the hydrolysis reactions, GC‐MS analysis was carried out (Figures S4 and S5). The MS results of TNPc degradation showed a peak at 173.0 (m/z) which corresponded well with 4‐nitrophthalonitrile. A peak at 144.0 (m/z) was also observed for TAPc degradation which was ascribed to 4‐aminophthalonitrile. The existence of carbonitrile group in the products is also evident from the obtained FTIR spectra; for example, a peak was observed at 2240 cm^−1^ for the hydrolysis product of TNPc. A similar peak (at 2255 cm^−1^) was observed in the hydrolysis product of TAPc (Scheme S4). ^1^H NMR and ^13^C NMR spectroscopy were used to further confirm the structures of the phthalonitriles (Figure S6 and S7). The NMR spectra of both the nitro‐ and amino−phthalonitriles are in agreement with known spectral data.^[22]^ An attempt to decompose an unsubstituted Pc was discouraged by solubility problems. However, it is worthwhile to note that the hydrolysis reaction is not selective to only phthalonitriles as there were many unexplained peaks observed in the MS spectrum of TAPc degradation products when recorded before the purification step, and it is thought that some of the products react with rhenium ions to form rhenium complexes. The yields for the phthalonitriles were, however, relatively high (58 and 64 %, respectively, for the amino and nitro derivatives).

The reaction seems to be rhenium(V)‐catalysed, since it only occurs in the presence of triphenylphosphine, which is known to reduce rhenium(VII) to rhenium(V) and is itself oxidized to triphenylphosphine oxide.^[23]^ The reaction also requires the presence of oxygen since it did not occur under a nitrogen atmosphere; hence, an oxidative hydrolysis of phthalocyanines is suggested. The mechanism of the hydrolysis of tetrasubstituted phthalocyanines under these conditions is still unclear. It was speculated that the hydrolysis might be taking place in multiple steps, even though there were no intermediate species isolated and characterized.

### Direct Metalation of Metal‐Free TAPc with Rhenium(V)

**Scheme 2:**
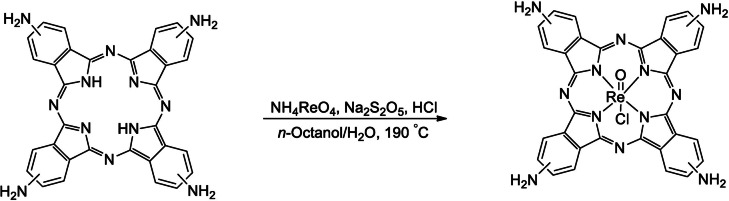
Direct synthesis of a rhenium(V)−phthalocyanine (Re−Pc) complex from tetraaminophthalocyanine (TAPc) and ammonium perrhenate in the presence of sodium metabisulfite as a reducing agent.

The synthesis of a Re^V^−Pc complex with an oxido ligand in the apical position is also reported in this account. This complex was successfully synthesized by direct metalation of metal‐free phthalocyanine using ammonium perrhenate and sodium metabisulfite as a reductant. The oxido‐metallates are already known with porphyrin as macrocyclic systems,^[6]^ and Ziener et al. reported the synthesis of the first oxido rhenium(V)−phthalocyanine complexes.^[9]^ The synthesis involves the reaction of rhenium pentachloride with phthalonitrile to yield the oxido rhenium−phthalocyanine complex and is thus not a direct metalation method. In this study, the oxido complex is obtained using ammonium perrhenate directly as a precursor and sodium metabisulfite (Na_2_S_2_O_5_) as a two‐electron reducing agent. This synthesis involves the metalation of a pre‐formed phthalocyanine instead of the one‐pot synthesis reported previously.^[9]^ The reaction was carried out in a mixture of *n*‐octanol and water (Scheme 2).

**Figure 5:**
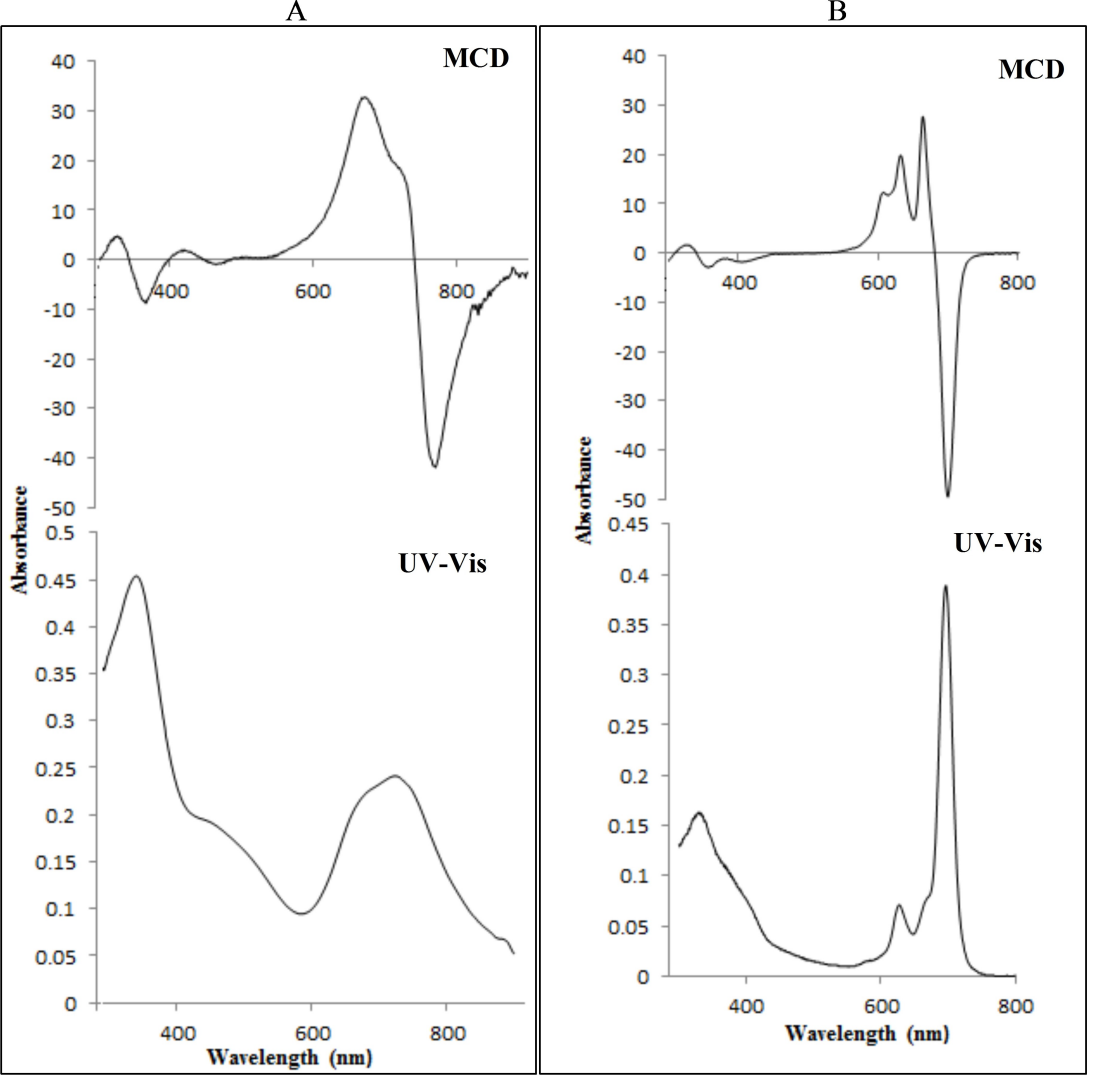
UV‐Vis and MCD spectra of (A) tetraaminophthalocyanine (TAPc) and (B) rhenium(V)−tetraaminophthalocyanine (Re−TAPc).

The success of this reaction was confirmed with electronic absorption techniques, that is, UV‐Vis and MCD. The UV‐Vis spectra of both metal‐free TAPc and Re−TAPc shows the B (soret) band at 360 nm and 318 nm, respectively (Figure 5). The UV‐Vis spectrum of TAPc does not clearly show a split Q band at 730 nm as expected for a metal‐free Pc instead a very broad Q band at 730 nm is observed due to aggregation. After metalation with rhenium, a relatively sharp Q band is observed at 694 nm suggesting that metalation of TAPc was achieved. MCD was used to confirm the UV‐Vis results.

MCD can be employed in the characterization of Pcs since it can also give information on electronic transitions, B(0,0) and Q(0,0) bands in the case of the Pcs, due to the presence of intense Faraday A_1_ terms or coupled pairs of oppositely‐signed Faraday B_0_ terms.^[24]^ The MCD spectra of TAPc and Re−TAPc (Figure 5) exhibited very intense B_0_ terms and the B_1_ and B_2_ bands lie in the 300–400 nm range. The major difference between the metal‐free and metalated Pc is visible in the Faraday A_1_ term, where a sharp negative A_1_ term appeared at 720 nm in the metalated Pc while a peak is observed with the unmetalated Pc at 775 nm. Positive A_1_ term is observed at 667 nm in Re−TAPc spectrum while the TAPc spectrum appears to have two bands in the A_1_ region at 684 and 728 nm. The FTIR spectra of TAPc and ReTAPc were very similar, and both show peaks at 3381 cm^−1^, 3245 cm^−1^ and 1612 cm^−1^ that can be ascribed to N−H stretching and bending vibrations of amine groups.

### Synthesis and Characterization of Rhenium Oxide Nanoparticles

The synthesis was carried out in aqueous solution (0.9 % saline), mimicking the rhenium conditions in a ^188^W/^188^Re generator. Sodium borohydride, NaBH_4_, was employed as a reducing agent (Scheme 1). During the synthesis of Re_x_O_y_ NPs, when NH_4_ReO_4_ was dissolved in saline solution together with an acetate buffer, the solution remained colourless; however, upon the addition of NaBH_4_, the solution changed to a tan colour. The observed colour change is consistent with the formation of colloids.^[25]^ A further colour change to black was observed when the nanoparticle solution was left at room temperature, and that has been attributed to the reoxidation of Re_x_O_y_ nanoparticles.^[26]^

**Figure 6:**
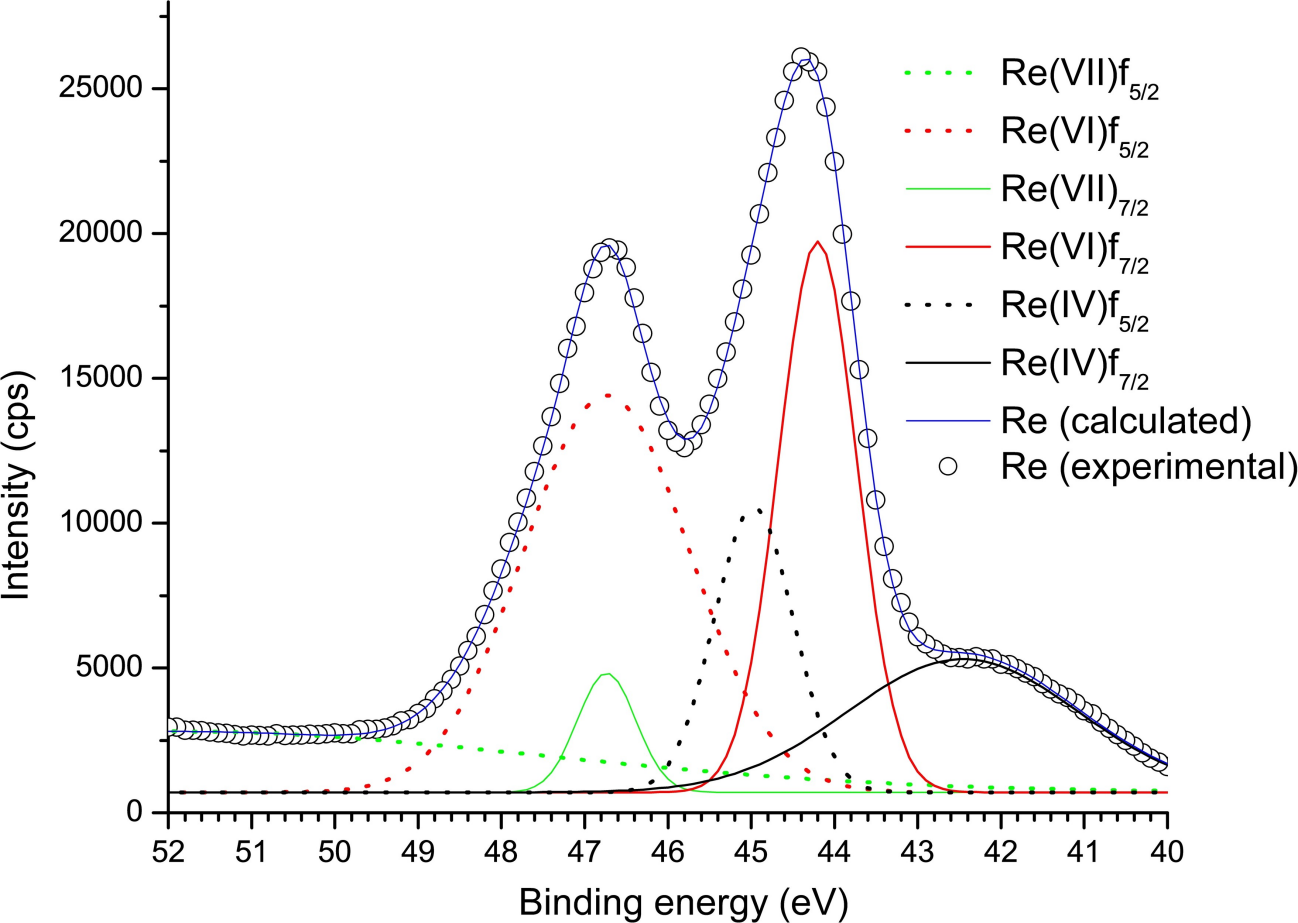
High‐resolution Re 4f XPS spectrum of the mixed valence rhenium in Re_x_O_y_−TAPc−FA NPs.

X‐ray photoelectron spectroscopy (XPS) and powder x‐ray diffraction spectroscopy (PXRD) analyses were carried out to investigate the rhenium nanoparticles′ oxidation state, phase purity and crystallinity. The reduction of perrhenate to a specific oxidation state is difficult to control, resulting in a mixture of rhenium oxides.[^27^, ^28^] Mucalo et al. also reported this problem[^29^, ^30^] and we also observed the presence of mixed valences (Figure 6). Previously, equilibrium phases of Re^IV^, Re^VI^, and Re^VII^ have been reported in one system.^[31]^ The XPS spectra showed a peak at 41.6 eV as expected for the Re4f_7/2_ state for the Re^4+^ species (Figure 6).^[32]^ The more intense peak around 41.6 eV is attributed to the Re4f_7/2_ state of the Re^6+^ species and the Re4f_5/2_ state of the Re^4+^ species as previously reported.^[33]^ The peak at 46.9 eV is due to the Re5f_5/2_ state of the Re^6+^ species and the Re 5f_7/2_ state of the Re^7+^ species.^[34]^ For the deconvoluted peaks, the Re 4 f doublets had the expected 2.4 eV multiplet splitting.[^35^, ^36^] The presence of the expected surface composition is provided in the wide scan spectrum (Figure S8A).

The N 1s XPS spectra confirm the presence of at least three N types (Figure S8B). The peak observed at 399.2 eV is attributed to aromatic N (−C−NH−C−) species, while the peak at 400.9 eV is attributed to amine N (−NH_2_) and aromatic N (−C=N−C−) species and the peak at 402.2 eV is attributed to amide N (−C(=O)−N) species.^[35]^ The obtained peak abundances are 19.5 %, 62 %, and 18.5 %, and the expected peak abundances of ReTAPc−FA nanoparticles had been calculated to 17 %, 66 % and 17 %. These values are relatively close to each other, and the slight difference can be attributed to the presence of the Re−N bond which was reported to be nitrogen‐containing compound dependent.[^37^, ^38^] The presence of phthalocyanine and folate nitrogen atoms is conclusive.

The C 1s spectra showed four peaks at 283.5, 284.0, 287, 288.9 and 290.6 eV (Figure S8C). The C 1s peaks at 283.5 and 284.9 eV are attributed to sp^2^ C−C/C−H, and sp^3^ C−C/C−H species, respectively. The 287 eV peak is attributed to the (C−NH_2_) carbon atom, while the 288.9 and 290.6 eV peaks are attributed to −C(=O)−NH and −C(=O)−OH, respectively. The obtained peak abundances of 70.3 %, 17.9 %, 7.9 %, and 4 % differ from the expected 87 %, 8 %, 3.6 %, and 1.8 % for C−C/C−H, C−NH_2_, CONH, and COOH, respectively. There was no direct interaction between carbon and rhenium as expected and evidenced by the absence of any binding energy shifts for metal carbides usually expected around 283 eV (Figure S8C).^[35]^ The O 1s XPS spectra showed an aromatic (C(=O)−NH peak at 531.1 eV and an aliphatic (C(=O)−NH) peak at 532.6 eV (Figure S8D). The −(C=O*)−OH and −C(=O)−O*H peaks were observed at 533.9 and 535.0 eV, respectively. The obtained abundances of 34.6 %, 34.9 %, 23.6 %, and 6.9 % differ from the expected 20 %, 40 %, 20 %, and 20 %. However, the XPS findings were conclusive on the expected atoms.

**Figure 7:**
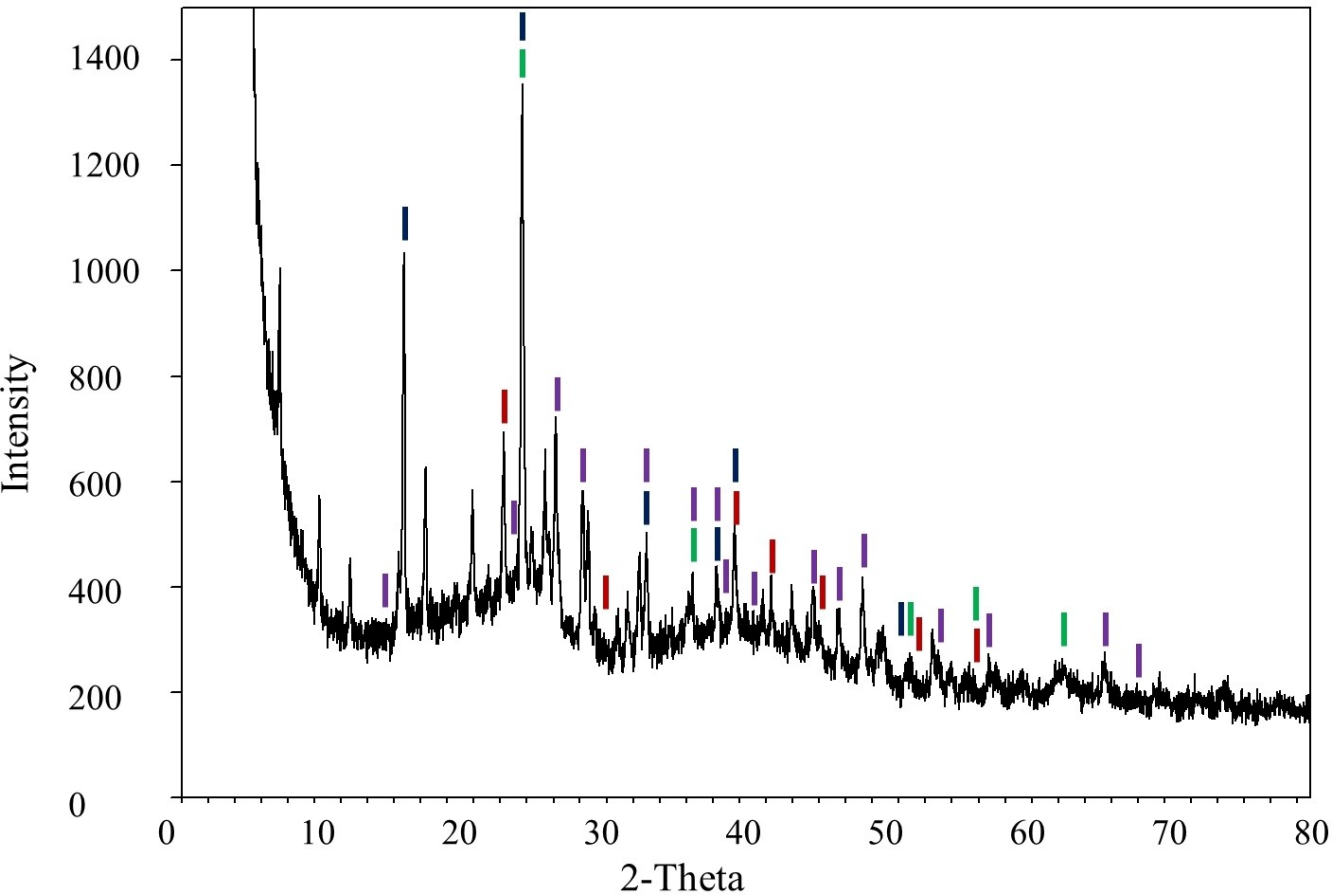
PXRD diffractogram of Re_x_O_y_−TAPc−FA NPs; ReO_3_ (red), ReO_2_ (green), NH_4_ReO_4_ (blue) and H_0.57_ReO_3_ (purple).

The PXRD analysis further confirmed the presence of Re^IV^, Re^VI^, and Re^VII^, corresponding to ReO_2_, ReO_3_, NH_4_ReO_4_ and H_0.57_ReO_3_, respectively (Figure 7).[^39^, ^40^, ^41^] However, the PXRD profiles (Figure 7) showed multiple phases of amorphous rhenium oxides as expected for mixed‐valence rhenium oxide nanoparticles.[^28^, ^42^]

### Folate−Tetraaminophthalocyanine‐Capped Nanoparticles (Re_x_O_y_−TAPc−FA NPs).

**Figure 8:**
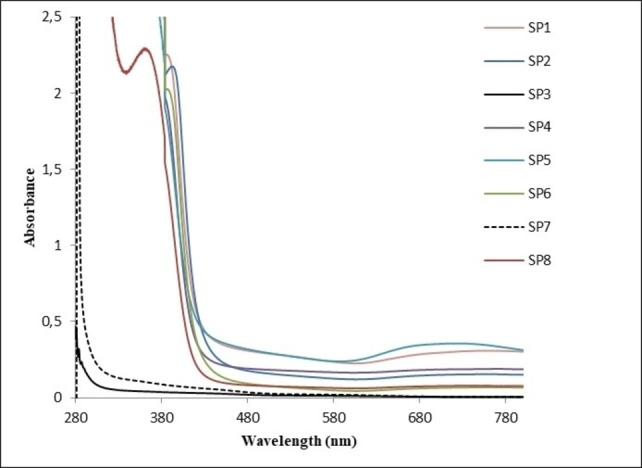
Optical spectra of folate‐conjugated tetraamine−phthalocyanine‐ capped (TAPc−FA) rhenium nanoparticles (Re_x_O_y_ NPs). Relative amounts of reagents used for the synthesis of **SP1**–**SP8** are described in Table S1.

**Figure 9:**
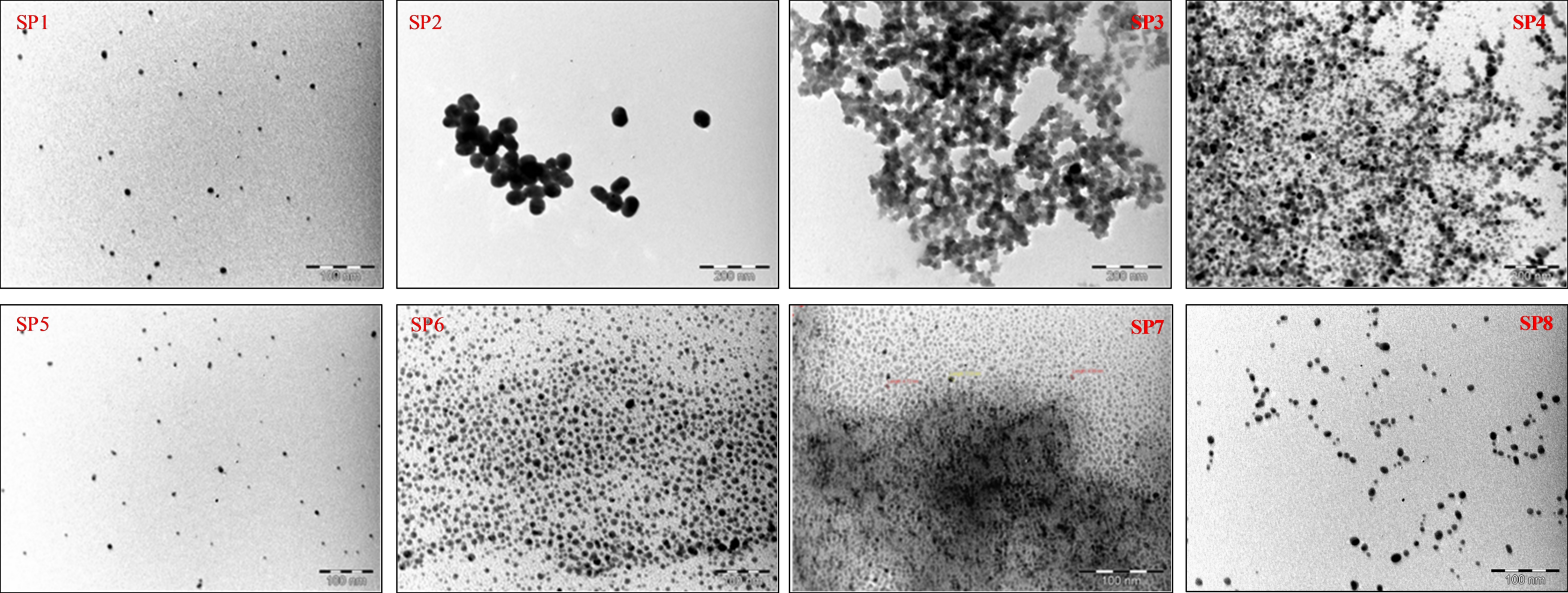
TEM micrograms of folate‐conjugated tetraaminophthalocyanine‐capped (TAPc−FA) rhenium nanoparticles (**SP1**–**SP8**).

In our initial synthesis, Re_x_O_y_−TAPc−FA NPs of broad size distribution were obtained. In order to obtain NPs of narrow size distribution, we optimized our synthesis by changing certain reaction parameters (**SP1**–**SP8**, Table S1). The UV‐Vis spectra of Re_x_O_y_−TAPc−FA NPs are shown in Figure 8. The surface plasmon resonance (SPR) peak of the Re_x_O_y_ NPs is observed around 380 nm (except for **SP3** and **SP7**). The peak is relatively narrow, indicating a narrow size distribution of the particles. The SPR peak tends to be red‐shifted when the amount of the metal salt increases and the amounts of the reducing and capping agent decrease. Another broad peak is observed at 712 nm corresponding to the Q band of the metal‐free TAPc capped with folic acid. The peak around 712 nm becomes more pronounced as the particle size increases. The UV‐Vis spectra of **SP3** and **SP7** do not show the SPR peak; this could be due to the size in **SP7** and reoxidation of **SP3** given the evidence of the formation of nanoparticles in the TEM micrograms obtained (Figure 9).

The SPR absorption of the Re_x_O_y_ NPs and their corresponding average particle sizes was documented (Table S2). The fluorescence properties Re_x_O_y_ NPs of sizes 10 nm and 50 nm were studied by spectrofluorimetry to provide information for possible investigation of their cell localization ability on cancer cells using confocal fluorescence microscopy studies. The emission spectra obtained for the two particle sizes were very similar (Figures S9A and S9B). To understand the fluorescence properties of the size 10 nm rhenium nanoparticles, a sample containing these NPs was irradiated at wavelengths ranging between 350 nm and 488 nm (Figure S10) for excitation. The resulting fluorescence emission spectra showed two emission peaks at 438 nm and 699 nm upon excitation at the wavelength of 350 nm. Excitation at 405 nm resulted in red‐shifted fluorescence emission spectra with maxima observed at 445 nm and 518 nm. The excitation at 488 nm also resulted in a red‐shifted peak at 570 nm; a difference of 132 nm from the 350 nm excitation wavelength. An excitation wavelength of 350 nm was selected for confocal fluorescence studies since it gave a narrow emission spectrum.

The spectra were recorded in water and two fluorescence emission peaks were observed at 438 nm and 699 nm, for both particle sizes. Compared to the 50 nm‐sized Re_x_O_y_ NPs, the 10 nm‐sized nanoparticles exhibited an increased fluorescence intensity. The peak at 699 nm is due to the Pc fluorescence since it was used as a capping agent (Figure S10). The fluorescence emission wavelength was kept at 580 nm whilst measuring excitation spectra. The fluorescence of the NPs showed a Stoke shift of 41 nm.

The synthesis procedures varied the relative amounts of metal content, capping and reducing agent amounts as illustrated in Table S1 to yield different synthesis products (SP) as presented in Table S2, and the spectroscopic data was further confirmed with transmission electron microscopy (TEM). The TEM micrograms of folate‐conjugated tetraaminophthalocyanine‐capped (TAPc−FA) rhenium nanoparticles (Re_x_O_y_−TAPc−FA NPs) are presented in Figure 9 with the preferred sizes for biological studies being 10 nm (SP2) and 50 nm (SP8) for size‐dependent folate‐receptor‐mediated endocytosis.

### Biochemical Evaluation of the Rhenium Oxide Nanoparticles

**Figure 10:**
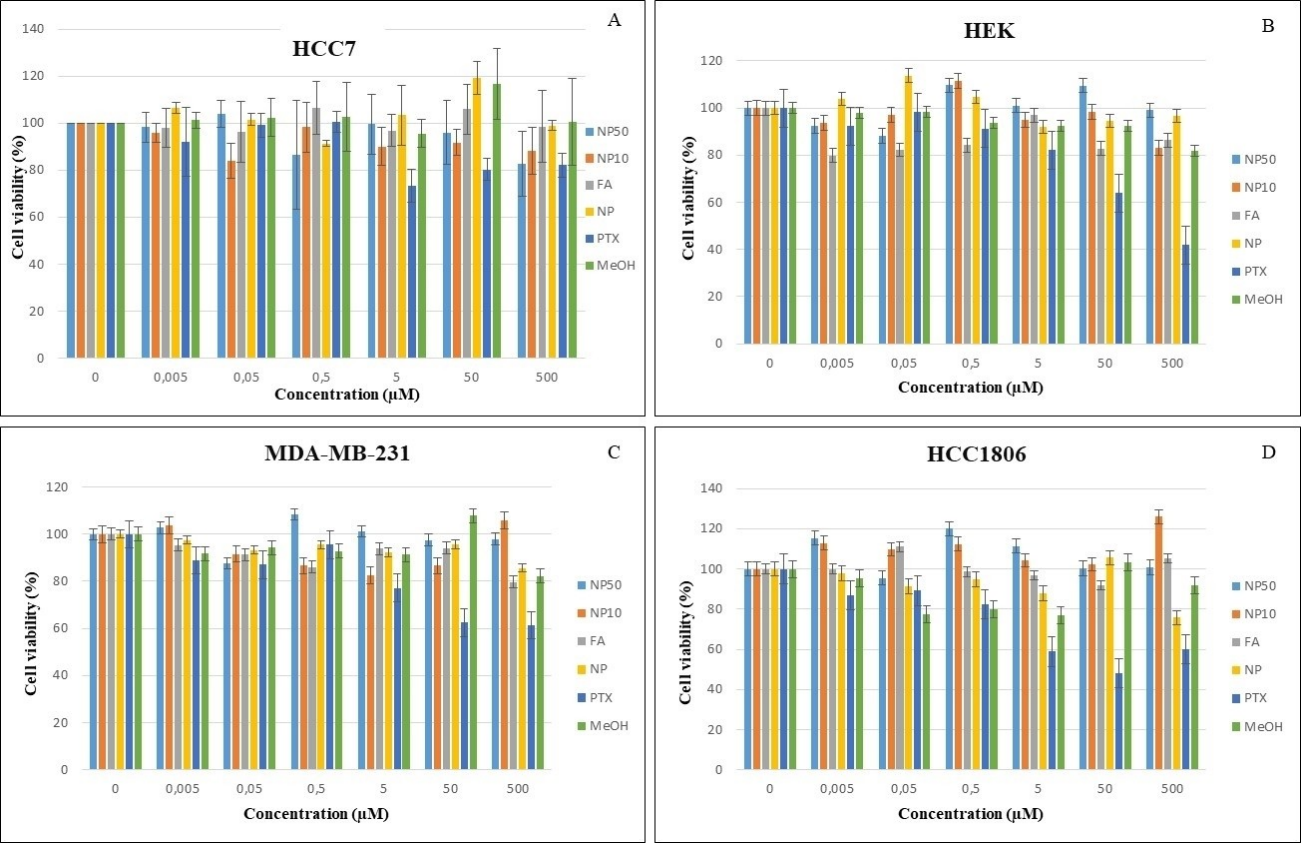
Cytotoxicity profiles of folate−tetraaminophthalocyanine capped rhenium oxide nanoparticles (Re_x_O_y_−TAPc−FA NPs) of sizes 10 nm (NP10) and 50 nm (NP50), folic acid (FA), borohydride capped rhenium nanoparticles (NP), paclitaxel (PTX) and methanol (MeOH) on (a) HCC7, (b) HEK, (c) MDA−MB‐231, and (d) HCC1806 cell lines.

Three breast cancer cell lines MDA−MB‐231, HCC70, HCC1806 with low, medium and high folate receptor levels (FR), respectively, and a non‐cancerous cell line model (HEK298T) were employed in the cytotoxicity studies. The aim was to establish the concentrations with high cell viability (i. e., non‐cytotoxic concentrations), since the cells will allegedly be destroyed by radiation as per the radiopharmaceutical development strategy. The viability profiles of dose‐dependent, size‐dependent and bioconjugation towards cytotoxicity evaluation of Re_x_O_y_ NPs are reported (Figure 10). The profiles of FA, methanol (the dispersion solvent), paclitaxel PTX (as the positive control) and untreated cell lines (as negative control) were investigated. Intracellular localization of 10 nm folate−tetraaminophthalocyanine‐functionalised rhenium nanoparticles (Re_x_O_y_−TAPc−FA NPs) in HCC1806 cells was examined and showed positive results (Figure 10).

### Cell Cytotoxicity Studies

The viability profiles of dose‐dependent, size‐dependent and bioconjugation towards cytotoxicity evaluation of Re_x_O_y_ NPs were obtained (Figure 10). The profiles of FA, methanol (the dispersion solvent), paclitaxel PTX (as the positive control) and untreated cell lines (as negative control) are also reported. For dose dependent responses, the cell survival rate of the different Re_x_O_y_ NPs obtained from varying concentrations (500, 50, 5, 0.5 and 0.005 μm) showed mixed results for all cell lines, but generally they show a slight decrease in cell viability with the increase in the concentrations of the compounds.

For bioconjugation dependent investigation, similar results were obtained for all cancer cell lines and the non‐cancerous control cell line upon exposure to TAPc−FA‐capped Re_x_O_y_ NPs. There is no clear pattern in the evaluation of the size‐dependent response of the two different sizes containing the same capping agent. Size‐dependent cytotoxicity studies of different nanoparticle systems have also been reported in the literature and the results are still unclear.^[43]^ Cell survival rate was more than 80 % for all the concentrations, meaning all the concentrations could be used for cell uptake studies (Figure 10).

### Cell Uptake Studies: Confocal Fluorescence Microscopy

**Figure 11:**
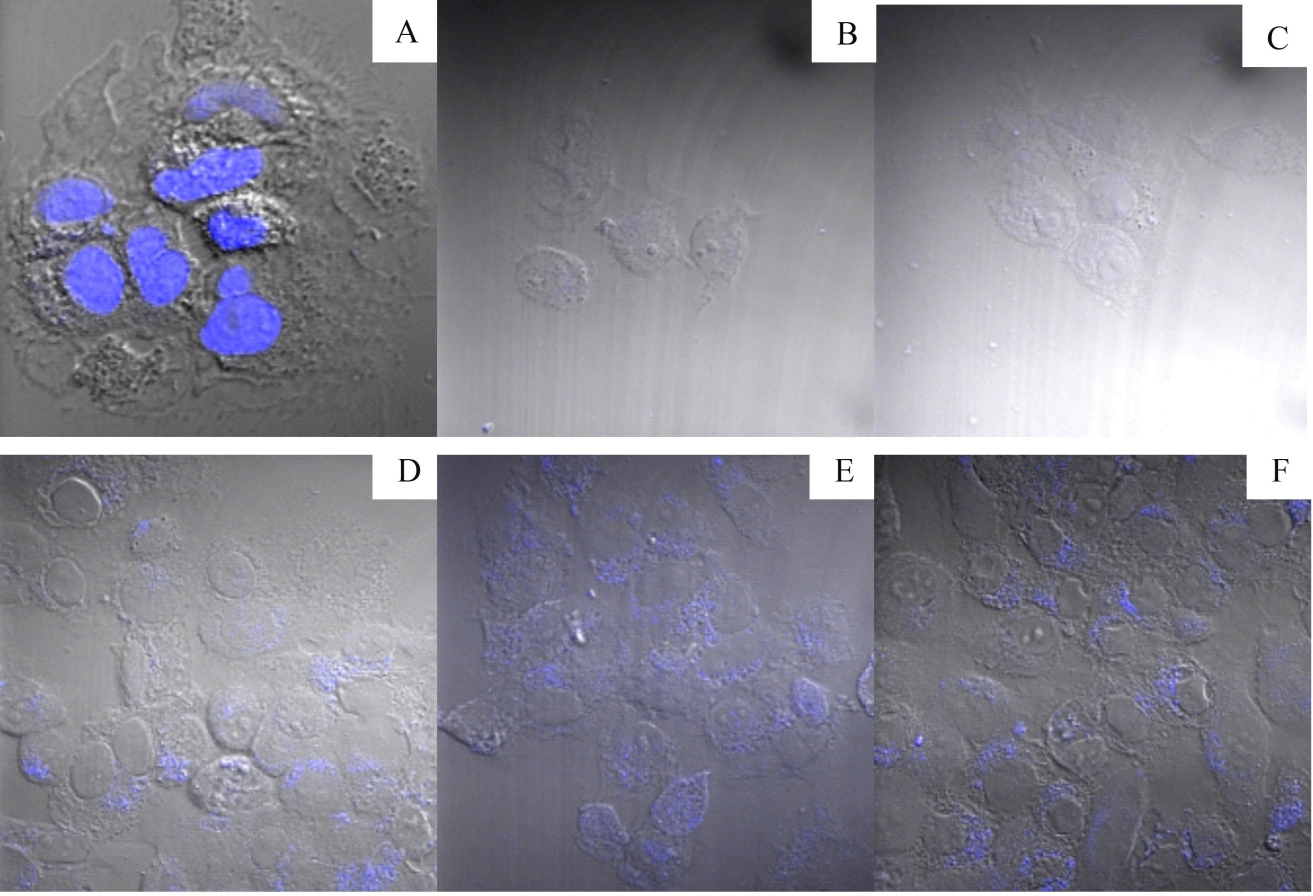
Confocal fluorescence images of HCC1806 cell lines treated with: (A) Hoechst 33324 nuclear stain, (B) folic acid, (C) methanol, (D) uncapped Re_x_O_y_ NPs, (E) 50 nm Re_x_O_y_ NPs and (F) 10 nm Re_x_O_y_ NPs. Blue colour indicates the signal from the treatment, while the cell outline is displayed as differential contrast.

The intensity of borohydride‐capped Re_x_O_y_ NPs (size 50 nm) was low relative to 50 nm TAPc−FA‐capped Re_x_O_y_ NPs, and the 10 nm particle size NPs exhibited the highest intensity in HCC1806 cell lines (Figure 11). This could be due to the particle size effect because particles of smaller sizes exhibit good fluorescence properties as compared to “bigger” nanoparticles of the same material. The high fluorescence intensity of the 10 nm NP‐treated cells could also be due to higher concentrations of size 10 nm NPs that are internalised in the cells.

To exclude the possibility that the NPs adhered to the surface of the cells and were not internalised, z‐stacks of the cells were performed using ZEN blue software. The z‐stack option sections the cell, to track exactly which depth of the cell exhibits the fluorescent nanomaterials. Confocal fluorescence microscopy showed that the nanoparticles were internalised since the fluorescence was only detected inside the cytoplasm, and the maximum intensity was observed at a depth of 12 μm (Table S3). A similar trend was observed for the uncapped Re_x_O_y_ NPs and for the folate‐conjugated 50 nm Re_x_O_y_ NPs (Figure 11).

### Cell Uptake Studies: Transmission Electron Microscopy

**Figure 12:**
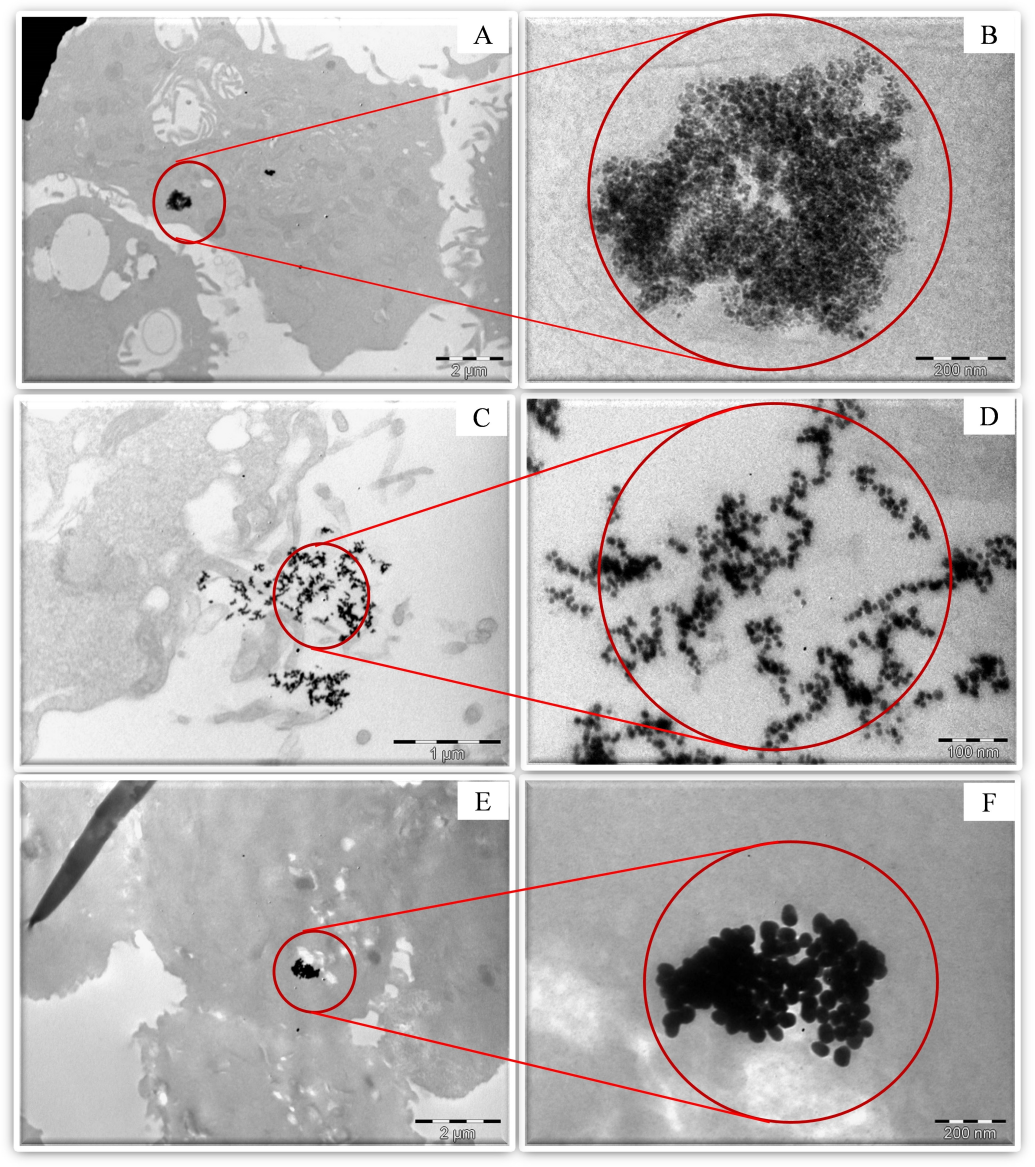
TEM images of the HCC1806 cells treated with (a) Re_x_O_y_−TAPc−FA NPs (50 nm) full view and (b) zoomed closer view, (c) Re_x_O_y_−TAPc−FA NPs (10 nm) full view and (d) zoomed closer view, and (e) borohydride‐capped Re_x_O_y_ NPs (50 nm) and (f) magnified view.

TEM images of untreated HCC1806 cells showed no morphological alterations and there are no nanoparticles observed as expected (Figure S11). The 10 nm Re_x_O_y_−TAPc−FA NPs exhibited better localisation patterns than the 50 nm‐sized NPs and the borohydride‐capped Re_x_O_y_ NPs as also observed in the confocal fluorescence studies. The 50 nm Re_x_O_y_−TAPc−FA NPs also showed particles of around 20 nm nanoparticles in addition to the 50 nm particles (Figure 12). This could be due to instability of the Re_x_O_y_ NPs under physiological conditions resulting in the breakdown of the particles. The 10 nm‐sized Re_x_O_y_ NPs also show quite a number of cells that they have not been internalised in (Figure 12), and while the cell lines for borohydride‐capped Re_x_O_y_ NPs were not properly fixed, they still showed tumour cell localisation to some extent (Figure 12). In general, the folate‐conjugated nanoparticles showed better internalization that the borohydride‐capped nanoparticles confirming the effect of the biomolecule (folate) for selective internalisation of the Re_x_O_y_ NPs by receptor‐mediated endocytosis.^[44]^

## Conclusion

The direct metalation of metal‐free tetrasubstituted nitro‐ or amino−metal‐free phthalocyanines with rhenium perrhenate in the presence of different two‐electron reducing agents was investigated. When PPh_3_ was employed as a reductant, the Pcs were hydrolysed to their subsequent nitro‐ or amino−phthalonitriles. The reaction seems to be catalysed by a rhenium(V) species formed upon PPh_3_ reduction of rhenium perrhenate. The reaction occurs in the presence of oxygen and not under a nitrogen atmosphere; hence an oxidative hydrolysis of phthalocyanines is suggested to take place. Furthermore, the desired Re^V^−Pc oxido complex was obtained from direct metalation of Pcs using perrhenate and sodium metabisulfite as the reducing agent. The success of the reaction was confirmed with spectroscopic techniques such as UV‐Vis and MCD. To the best of our knowledge, this work represents the first‐ever synthesis of Re^V^−Pc oxido complex through a direct metalation of H_2_Pcs with perrhenate in the presence of metabisulfite. It is worth noting that further optimization of the synthesis to improve the yield and a single‐crystal X‐ray characterization of the complex is still needed. Attempts to grow single crystals suitable for X‐ray diffraction analysis have thus far been thwarted by the poor solubility of the complex in most solvents; hence, crystal growth was unsuccessful.

The synthesis of Re_x_O_y_ NPs from the reduction of perrhenate with NaBH_4_ was carefully optimized by varying reaction parameters, allowing the synthesis of NPs with different sizes (between 10 nm and 50 nm). Thus, stable folate−tetraaminophthalocyanine‐functionalised rhenium oxide nanoparticles (Re_x_O_y_−TAPc−FA NPs) were synthesized from perrhenate using sodium borohydride as a reducing agent, and they were confirmed to have variable oxidation states by XRD and XPS. Cytotoxicity investigation of the Re_x_O_y_−TAPc−FA NPs (10 nm and 50 nm) on HCC7, HCC1806, HEK and MDA−MB‐231 cell lines showed mixed results for all the cell lines. Still, generally, they showed a slight decrease in cell viability with the increase in the concentrations of the test materials. A cell survival rate above 80 % was maintained for all the cell lines at the investigated concentrations. Cell uptake studies were also conducted on the HCC1806 cell line using confocal fluorescence microscope, and the results obtained were confirmed with TEM. Generally, the nanoparticles were internalised with a size of 10 nm nanoparticles showing better results than 50 nm while borohydride‐capped Re_x_O_y_ NPs showed poor cell uptake results. Folate functionalisation showed enhanced cellular uptake confirming the prospective applications of folate‐functionalised rhenium oxide nanoparticles for cancer targeting, suggesting that the targeting biomolecule (FA) does enhance cell accumulation of the Re_x_O_y_ NPs. This work contributes to the cell uptake studies of rhenium oxide nanoparticles.

## Experimental Section

Single crystals of the product of the decomposition of tetranitrophthalocyanine (TNPc), which were suitable for X‐ray analysis, were obtained by the slow evaporation of the mother liquor of the synthetic solution. Unfortunately, single crystals of the monomeric rhenium(V) complex, suitable for X‐ray analysis, could not be obtained. Single‐crystal X‐ray diffraction (SCXRD) studies were performed with a Bruker Kappa Apex II diffractometer equipped with graphite‐monochromated Mo K_α_ radiation, *λ*=0.71073 Å. Intensity data collection was obtained at 200 K using the ω‐2θ scan technique. Data was collected using APEX‐II, and cell refinement and data reduction were carried out using Bruker SAINT.^[45]^ The structure was solved through direct methods using SHELXS‐2013^[46]^ and subsequently refined by least‐squares procedures using SHELXL‐2013 equipped with SHELXLE^[47]^ as a graphical interface. Data were corrected for absorption effects using the numerical method implemented in SADABS.^[45]^ The summary of crystal data, data collection and structural refinement parameters for 4‐nitrophthalonitrile are given in Table 2. Deposition Number 1406176 (for 4‐nitrophthalonitrile) contains the supplementary crystallographic data for this paper. These data are provided free of charge by the joint Cambridge Crystallographic Data Centre and Fachinformationszentrum Karlsruhe Access Structures service.

**Table 2:** Crystal and structure refinement data of 4‐nitrophthalonitrile.

Compound	4‐Nitrophthalonitrile
Empirical formula	C_8_H_3_N_3_O_2_
Formula weight (M)	173.13
Temperature, *T* (K)	200 K
Wavelength, Mo K_α_ (Å)	0.71073
Crystal system	Orthorhombic
Space group	*Pbca*
*a* (Å)	12.9000(5)
*b* (Å)	9.2880(5)
*c* (Å)	13.3068(6)
Volume, V (Å^3^)	1594.36(13)
Z	8
Calculated density, *ρ* (Mg m^−3^)	1.443
Absorption coefficient, *μ* (mm^−1^)	0.109
F(000)	704
Crystal size (mm)	0.322×0.348×0.400
Index ranges	−17≤h≤11, −12≤k≤12, −17≤l≤17
Reflections collected	21572
Independent reflections	1985
Completeness to θ= 28.00°	99.8 %
Data/restraints/parameters	1985/0/118
Goodness‐of‐fit on *F* ^*2*^	1.043
*R* indices	*R*_1_=0.0332, *wR*_2_=0.0972
Largest diff. peak and hole (eÅ^−3^)	0.25 and −0.21

### Rhenium(V)‐mediated hydrolysis of TNPc

**Scheme 3:**
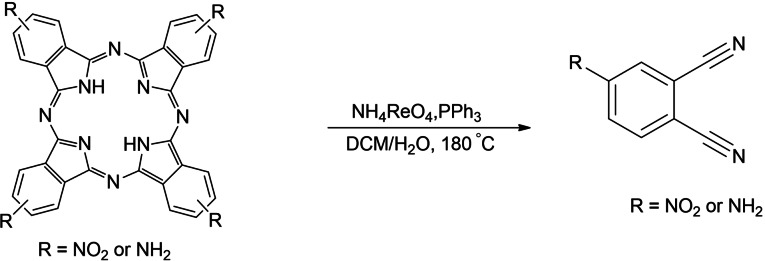
Phthalonitriles obtained in the degradation of R_4_Pcs using ammonium perrhenate in the presence of PPh_3_ as the reducing agent.

To a solution of TNPc (1 g, 1.44 mmol) and ammonium perrhenate (0.42 g, 1.57 mmol) in DCM (5 mL), was added a solution of triphenylphosphine (0.41 g, 1.57 mmol) in H_2_O (2 mL) and the mixture was heated at 180 °C for 4 h (Scheme 3). The reaction mixture turned from a dark green colour, a characteristic of Pcs, to a yellow colour. The solution was analysed using UV‐Vis spectrophotometry and GC‐MS. Single crystals also formed from this solution after three weeks of standing at room temperature, and these were analysed using single‐crystal X‐ray diffraction (SCXRD). In the hydrolysis of TNPc, the results were; Yield=64 %, Mp=142 °C. ^1^H NMR (400 MHz, DMSO‐d_6_) δ (ppm): 8.45 (d, C(15)−H), 8.67 (d, C(16)−H), and 9.03 (s, C(12)−H). ^13^C NMR (101 MHz, DMSO‐d_6_) δ (ppm): 115.38 (C(1)−N(2), 115.07 (C(2)−N(3)), 150.25 (C(11)−(N(1)), 117.10 C(13)), 120.72 C(14)), 129.33 C(12), 129.02 C(16)), 136.15 (C(15) (see the crystal structure for the numbering). IR (ν max, cm^−1^): 3102 and 3049 (aliphatic C−H), 2240 (C≡N), 1536 (N−O, asymmetric), 1353 (N−O, symmetric), 1608 and 1586 (aromatic C=C), 1300 (C−N). GC‐MS (m/z) [M+H]^+^: Found for C_8_H_3_N_3_O_2_=173.0, Calculated mass=173.13.

### Rhenium(V)‐Mediated Hydrolysis of TAPc

The hydrolysis of TAPc was carried out in the same manner as described for TNPc hydrolysis, and the following results were obtained (Scheme 3). Yield=58 %, Mp=180 °C. ^1^H NMR (400 MHz, DMSO‐d_6_) δ (ppm): 7.89 (s, 1H), 7.63 (d, 1H), 7.57 (d, 1H) and 3.43 (d, 2H).^13^C NMR (101 MHz, DMSO‐d_6_) (ppm): 129.28 (C(1)−N(2), 129.17 (C(2)−N(3)), 133.73 (C(11)−(N(1)), 131.90 C(13)), 132.00 C(14)), 132.59 C(12), 132.48 C(16)), 132.71 (C(15). IR (ν max, cm^−1^): 2965 and 2923 (N−H), 2851 (shoulder band), 2255 (C≡N), 1718 (N−H), 1262 (C−N), 721 (N−H). GC‐MS (m/z) [M+H]^+^: Found for C_8_H_5_N_3_=144.0, Calculated mass=143.03.

### Direct Metalation of Metal‐Free TAPc with Rhenium(V)

To a solution of 1 m HCl (80 μL) in 4 : 1 *n*‐octanol/water was added TAPc (100 mg, 0.17 mmol) and the mixture was stirred at 190 °C for 3 min. Subsequently, ammonium perrhenate (50 mg, 0.18 mmol) and sodium metabisulfite (32.3 mg, 0.17 mmol) as a reducing agent were added, and the reaction mixture was allowed to continue stirring for 4 h (Scheme 2). Thereafter, the reaction mixture was then cooled to room temperature and diluted with ethanol (20 mL), and the precipitate that resulted was collected by centrifugation 6400 rpm for 30 min. The solid was filtered and washed with water, methanol/diethyl ether (1 : 9), and then ethyl acetate/hexane (2 : 1). The solid was filtered and washed with water, methanol/diethyl ether (1 : 9), and then ethyl acetate/hexane (2 : 1). The yield was 20 % (2.75 mg). IR (cm^−1^): 3381, 3245, 1612 (N−H). UV‐Vis (nm): 694 and 318. MCD (nm): 720, 667, 376, 335 and 358.

### Synthesis of Rhenium Oxide Nanoparticles

The synthesis of rhenium oxide nanoparticles (Re_x_O_y_ NPs) (Schemes 1 and S3) was achieved as follows: To a solution of ammonium perrhenate (100 mm), acetate buffer (2.0 mL) and 450 μL saturated solution of saline (0.5 mL) was added a 100 mm ammonium perrhenate (80 μL) solution, the resulting mixture was allowed to stir under nitrogen for 30 min at room temperature. After the 30 min elapsed, a freshly prepared saturated solution of sodium borohydride (NaBH_4_) (0.4 mL) was added dropwise to the reaction mixture over a minute, during which the solution turned from colourless to tan. Then, a solution of TAPc−FA (10 mg mL^−1^) in dimethylsulfoxide (DMSO) (10 mg mL^−1^) was added slowly. The solution was vigorously agitated under a nitrogen atmosphere for a further 30 min. The solution was transferred to polyethylene tubes and was subjected to centrifugation, followed by filtration. The NPs in solution and were fixed for TEM analysis. The images were acquired from random areas, and ImageJ^TM^ software was used to calculate nanoparticle size distribution. To afford nanoparticles of different sizes, the reaction conditions were optimised using three variable chemometrics model (2^3^), where conditions such as the amount of the reducing agent (NaBH_4_), metal salt (NH_4_ReO_4_) and the capping agent (TAPc−FA) were varied systematically (Table S1). The bare nanoparticles (Re_x_O_y_ NPs) were also synthesised, and our understanding is that they are capped by borohydride ions.

## Conflict of interest

The authors declare no conflict of interest.

1

## Supporting information

As a service to our authors and readers, this journal provides supporting information supplied by the authors. Such materials are peer reviewed and may be re‐organized for online delivery, but are not copy‐edited or typeset. Technical support issues arising from supporting information (other than missing files) should be addressed to the authors.

Supporting Information

## Data Availability

The data that support the findings of this study are available in the supplementary material of this article.
