# Supplementary material for: Reaction of Perrhenate with Phthalocyanine Derivatives in the Presence of Reducing Agents and Rhenium Oxide Nanoparticles in Biomedical Applications
Source: ChemistryOpen. 2022 Jul 20;11(7):e202200037. doi: 10.1002/open.202200037 (PMC9297772; doi:10.1002/open.202200037)
Supplement: Supplementary file 1 — Supporting Information [file OPEN-11-e202200037-s001.pdf]

ChemistryOpen

Supporting Information

Reaction of Perrhenate with Phthalocyanine Derivatives in the Presence of Reducing Agents and Rhenium Oxide Nanoparticles in Biomedical Applications

Songeziwe Ntsimango,* Sendibitiyosi Gandidzanwa,* Sinelizwi V. Joseph, Eric C. Hosten, Marvin Randall, Adrienne L. Edkins, Samson M. Khene, Philani Mashazi, Tebello Nyokong, Abubak'r Abrahams, and Zenixole R. Tshentu*

Experimental Section

Materials and instrumentation methods

4-Nitrophthalonitrile (99%), 1,8-diazabicyclo[5.4.0]undec-7-ene (DBU) (98%), ammonium perrhenate (≥99%), and sodium sulfide nonahydrate (98%) were purchased from Sigma-Aldrich. Zinc chloride and triphenylphosphine (PPh_3) were obtained from Merck, while pyridine hydrochloride (98%) was sourced from Alfa Aesar, and sodium metabisulphite (98%) from ACE Chemicals. Solvents were obtained from Sigma-Aldrich and used as received. MDA-MB-231, HCC7, HCC1806 and HEK293T cell lines were purchased from American Type Culture Collection.

The UV-Vis absorption spectra were recorded on a Perkin Elmer UV-Vis spectrophotometer in the wavelength range 300-900 nm using DMSO/water as a solvent system. Magnetic circular dichroism (MCD) spectra were measured on a Chirascan Plus spectropolarimeter equipped with a permanent magnet that produces a magnetic field of 1 T (1 tesla). FT-IR spectra (4000−400 cm^{-1}) were run on a Bruker Tensor 27 platinum ATR-FTIR spectrometer. ^{1}H NMR and ^{13}C NMR spectra were recorded on a Bruker Avance AV 400 MHz spectrometer operating at 400 MHz for ^{1}H and 101 MHz for ^{13}C using DMSO-d_6 as solvent and tetramethylsilane as internal standard. The centrifugations were performed on a Labofuge Centrifuge 200 using 50 mL polyethylene tubes. The various products obtained upon hydrolysis of Pcs were confirmed using an Agilent 7890A gas chromatograph-mass chromatography (GC-MS) fitted with a 30 m x 0.25 mm x 0.25 µm DB-5 capillary column. The analysis run was started with an oven temperature of 50 °C ramping to 250 °C at 15 °C min^{-1} and helium was used as carrier gas at a flow rate of 1.6 $mL.min^{-1}$ with an average velocity of 30.2 cm s^{-1} and a pressure of 63.7 KPa. The Powder X-ray diffraction (PXRD) analysis of the nanoparticles was carried out at room temperature on a Bruker D2 powder x-ray diffractometer using Cu radiation (λ = 1.5406 Å) with a Lynxeye detector. A scan range from 5 to 100º 2θ at 0.02 steps and 0.2 sec/step. The phases in the samples were identified by comparing the diffraction pattern with known diffraction patterns in the Bruker EVA software library.

X-ray photoelectron spectroscopy (XPS) was conducted using a Kratos Axis Ultra DLD, with the Al (monochromatic) anode, equipped with a charge neutraliser. For wide XPS scans, the following parameters were used: emission 10 mA, anode (HT) 15 kV, operating pressure below 5 × 10^{-9} Torr, hybrid lens, and resolution to acquire scans was at 80 eV pass energy inslot mode. The centre used for the scans was at 597.5 eV and the width at 1205 eV, with steps at 1 eV and dwell time at 100 ms. The resolution was changed to 40 eV pass energy in slot mode for the high-resolution scans. Centre was at 288.5 eV and width at 23 eV for C 1s, with step size at 0.1 eV and dwell time at 500 ms. For the quantitative analysis, the area under the peaks was used for the atomic percentages obtained from elements present on the survey spectra and then deconvoluted. The binding energy was corrected against the adventitious carbon at 284.9 eV. NIST XPS Database [1] was used to identify the peaks and their oxidation state correctly.

Syntheses methods

Synthesis of tetraaminophthalocyanine (TAPc)

Tetraaminophthalocyanine (TAPc) was synthesized using a published method [2]. It was accomplished in three steps, namely, (i) synthesis of tetranitro-zinc-phthalocyanine (TNZnPc), (ii) reduction of tetranitro-zinc-phthalocyanine to tetraamino-zinc-phthalocyanine (TAZnPc), and (iii) demetalation of zinc to form free-base tetraaminophthalocyanine (**Scheme S1**). The synthesis of TNZnPc was based on a reported method [2], with a few modifications. 4-Nitrophthalonitrile (5.14 g, 29.7 mmol) and zinc chloride (1.23 g, 7.42 mmol) were added into *n*-octanol (8 mL), and catalytic amounts of 1,8-diazabicyclo[5.4.0]undec-7-ene (DBU) were added into the reaction mixture. The reaction was allowed to proceed at 180 °C for 4 h. Thereafter, the reaction mixture was left to cool to room temperature and diluted with toluene (80 mL). The precipitate that resulted was collected by centrifugation. The solid was filtered and washed with toluene, water, methanol/diethyl ether (1:9), and then ethyl acetate/hexane (2:1). TNZnPc was obtained as a dark green solid (5.08 g, 92%).

TAZnPc was synthesized under a nitrogen atmosphere at 70 °C by dissolving TNZnPc (4.08 g, 5.35 mmol) of TNZnPc in 80 mL dimethylformamide (DMF), followed by addition of sodium sulfide nonahydrate (15.88 g, 66.1 mmol). The reaction mixture was allowed to stir overnight. Upon completion, the mixture was then cooled to room

temperature and diluted with cold water (200 mL). A precipitate formed and was collected by centrifugation. The solid was repeatedly washed with methanol/diethyl ether (1:9), ethyl acetate and dried to afford TAZnPc as a dark green solid (2.46 g, 72%).

TAZnPc (1.50 g, 2.35 mmol) was dissolved in a solution of pyridine (4 mL) and pyridine ･HCl (2.03 g, 17.3 mmol) while being stirred under nitrogen at 110 °C for 17 h. Upon completion, the reaction mixture was diluted with water (20 mL) and centrifuged. The collected dark green precipitate was filtered and repeatedly washed with water, MeOH and EtOAc, and dried under vacuum to obtain a green solid, TAPc (1.25 g, 93%). TNZnPc was also demetalated similarly to TAZnPc to form TNPc with a yield of 85%.

Synthesis of folate-conjugated TAPc (TAPc-FA)

The synthesis of folate-conjugated tetraaminophthalocyanine (TAPc) was achieved according to a literature method (**Scheme 2**) [3], with the following minor adjustments; folic acid (200 mg, 0.35 mmol) was added to a solution of *N,N′*-dicyclohexylcarbodiimide (DCC) (7.0 mg, 0.39 mmol) and *N*-hydroxysuccinimide (NHS) (39 mg, 0.39 mmol) in DMSO and H_2O (1:1 v/v) solvent mixture. The resulting solution was stirred at room temperature for 24 h. TAPc (233 mg, 0.35 mmol) in DMSO was added to the solution and stirred for 48 h (**Scheme S2**). The resulting solution was centrifuged at 7000 rpm for 10 min and filtered, giving a green solid (0.22 g, 61%). IR (cm^{-1}): 3336 (N-H), 2935, 2853 (C=C) and 1694 (C=O). UV-Vis (nm): 740, 358 and 298. MCD (nm): 664 and 763. LC-MS (m/z) $[M+2H]^+$: Found for $C_8H_5N_3$ = 1017.82 $[M + 2H]^+$, expected mass $[M]^+$ = 1015.21

Biochemical studies

Three breast cancer cell lines, MDA-MB-231, HCC70 and HCC1806, with low, medium and high folate receptor expression, respectively, were cultured in Dulbecco's Modified Eagle Medium (DMEM) supplemented with 5% (v/v) heat-inactivated FCS, 1 mM L-Glutamine, 100 U/mL penicillin and 100 µg/mL streptomycin (Pen/Strep) at 37 °C in a humidified 9% CO_2 incubator [4]. The immortalized non-cancerous HEK293T cell line was maintained using a 1:1 ratio of Ham's F10 and DMEM supplemented with 5% (v/v) heat inactivated FCS, 100 U/ml penicillin and 100 µg/mL streptomycin

(Pen/Strep), 20 ng/mL epidermal growth factor (EGF), 100 ng/mL cholera toxin, 500 ng/mL hydrocortisone and 10 μg/mL insulin.

Cell proliferation assay

Cell viability studies in the presence of nanoparticles was evaluated by MTT assay according to manufacturer's instructions. The cells were seeded at 6,000 cells per well in a 96-well plate and allowed to settle overnight. The cells were treated with a range of concentrations (0, 0.5, 5, 50, 250 and 500 μM) of the two particle sizes (10 nm and 50 nm) of folate-tetraaminophthalocyanine (TAPc-FA) capped Re_xO_y NPs (Re_xO_y NP-TAPc-FA), FA or methanol (MeOH) vehicle control (0.02 % v/v MeOH) for 96 hours and absorbance at 595 nm recorded using a Powerwave spectrophotometer (BioTek). The half-maximal inhibitory concentration (IC_{50}) for each compound was calculated relative to the vehicle-treated control from a dose-response curve (log concentration vs absorbance at 595 nm) using non-linear regression with GraphPad Prism 4 (GraphPad Inc. Paclitaxel (PTX) was included as a positive control (IC_{50} of 100 nM). All treatments were conducted in triplicate on each of the two plates.

Cell-uptake studies: Confocal fluorescence microscopy

The cell-uptake studies were conducted using the HCC1806 (the cells with the highest folate overexpression) to verify the cellular localisation of the nanoparticle systems. HCC1806 cells were seeded on to sterile coverslips and permitted to settle and grow over 24 h before treating with 0.5 μM FA-TAPc capped Re_xO_y NPs (sizes 10 nm and 50 nm) and uncapped Re_xO_y NPs (50 nm), FA, MeOH and Hoechst 33342 overnight (as a positive control for staining of the nucleus). Subsequently, the cells were washed in pre-warmed bovine serum albumin (BSA) (2% in phosphate-buffered saline) and mounted on slides using DAKO antifade mounting medium. The Zeiss LSM 510 meta laser scanning confocal microscope was used to capture cell images containing the relevant compounds or Hoechst that were excited at 350 nm and emission detected in the 450/50 channel. The results were analyzed using Zen software (blue edition, Zeiss, Germany) or AxiovisionLE 1.4.7 (Carl Zeiss Imaging Solutions Germany).

Cell uptake studies: Transmission electron microscope

The transmission electron microscope (TEM) was employed to verify the results obtained with confocal fluorescence microscope. The same procedure described in

previously was followed. Preparations of the cells for viewing under TEM was carried out as follows; 2.5 M glutaraldehyde was added to the cells and refrigerated at 5 °C for 60 h. After 60 h, the cells were washed twice with 0.1 M sodium phosphate buffer over 10 min. Osmium tetroxide was added to cells, and left for 90 min at room temperature. After 90 min, the cells were washed with 0.1 M sodium phosphate buffer twice for 10 min. Subsequently, the cells were subjected to a series of washing with different solvents. Afterwards, the cells were suspended in a pure resin for 720 min, pure resin was added again into the cells and the cells were placed in an oven at 600 °C for 36 h to solidify the resin. The cell-containing solidified resin was fashioned into pellets and ultra-microtome was used to section the cells (embedded inside the pellets) into the thickness of 80 nm, at the cutting speed of 0.7 mm/sec, using a diamond knife. The sections were then viewed under TEM.

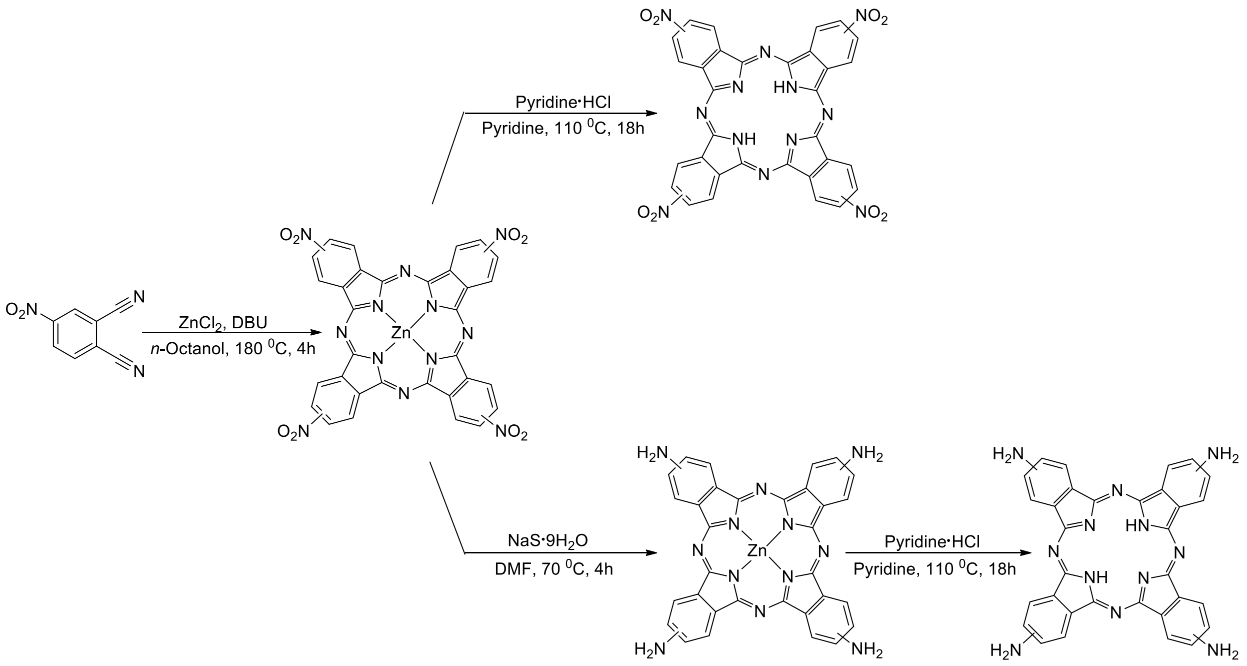

Scheme S1: The synthetic route of tetraaminophthalocyanine.

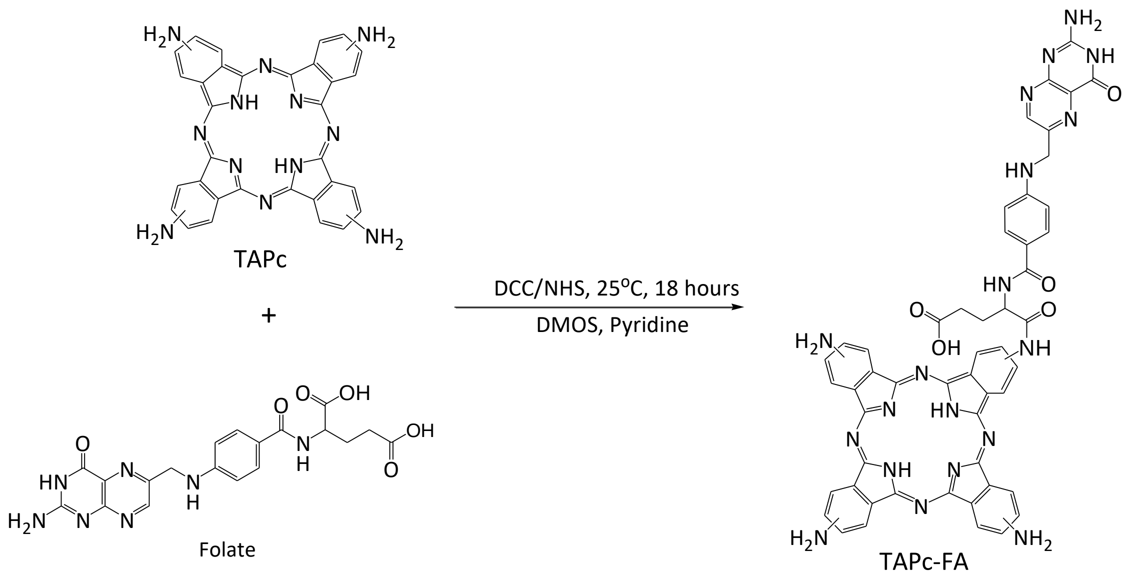

Scheme S2: Conjugation of tetraaminophthalocyanine (TAPc) with folate (FA). The γ-isomer is shown above, and the α-isomer is shown below in the formation of α-Re_xO_y-TAPc-FA NPs.

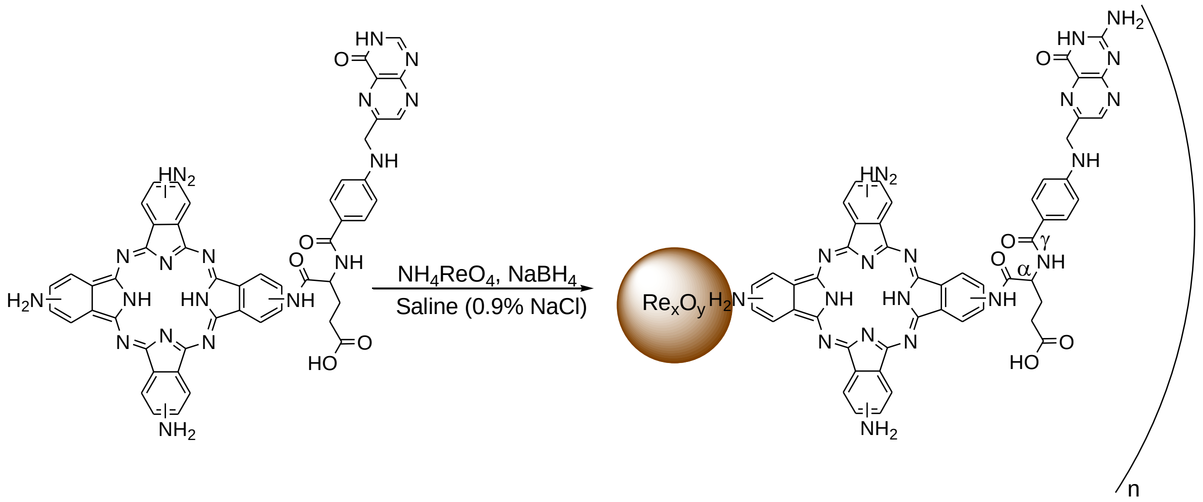

Scheme S3: The synthesis of Re_xO_y-TAPc-FA α-isomer from α-TAPc-FA in the presence of sodium borohydride as a reducing agent.

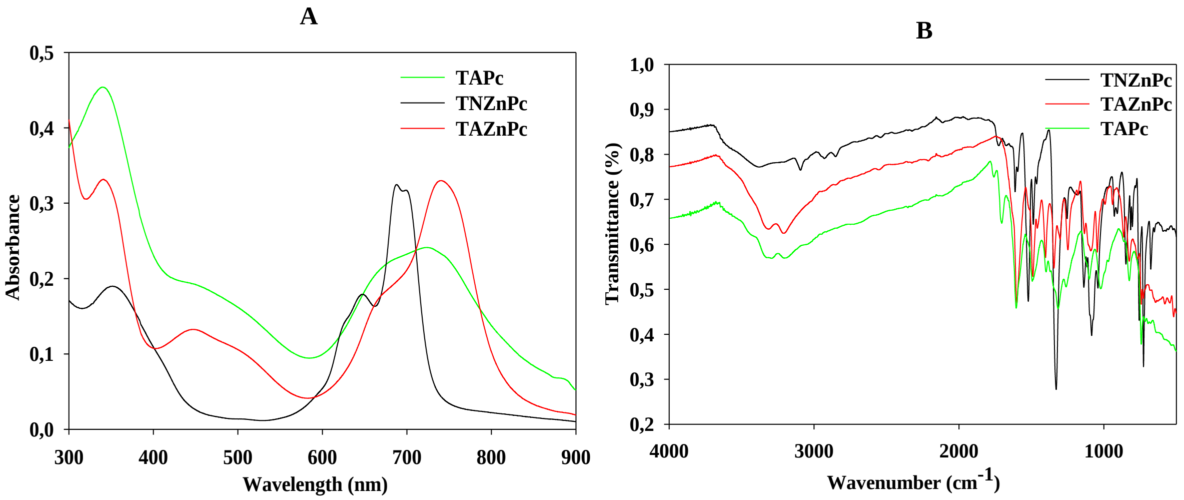

Figure S1:The spectra of tetranitro-zinc-phthalocyanine (TNZnPc), tetraamino-zinc-phthalocyanine (TAZnPc) and tetraaminophthalocyanine (TAPc): (a) UV-Vis, and (b) FT-IR

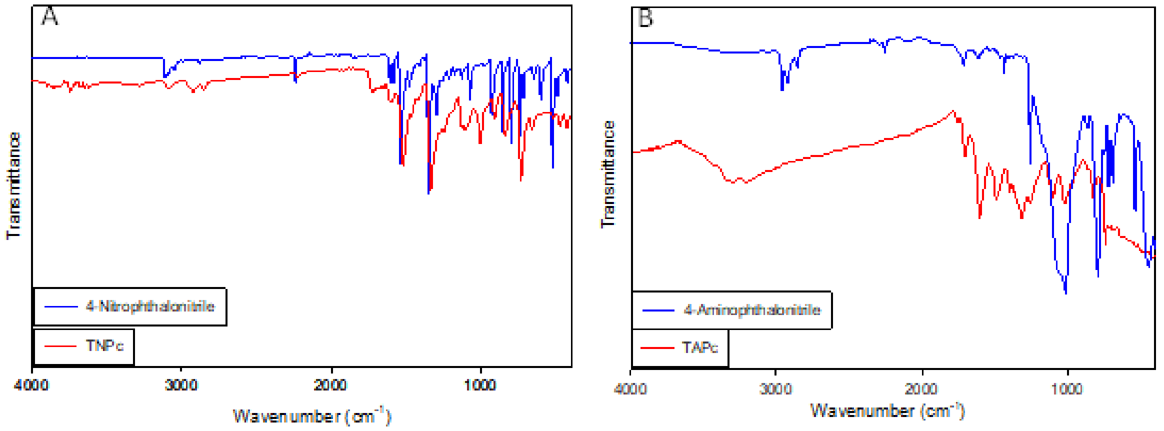

Figure S2: FT-IR spectra of; (A) tetranitro-phthalocyanine (TNPc) and the hydrolysis product (4-nitrophthalonitrile), and (B) tetraaminophthalocyanine (TAPc) and the hydrolysis product (4-aminophthalonitrile).

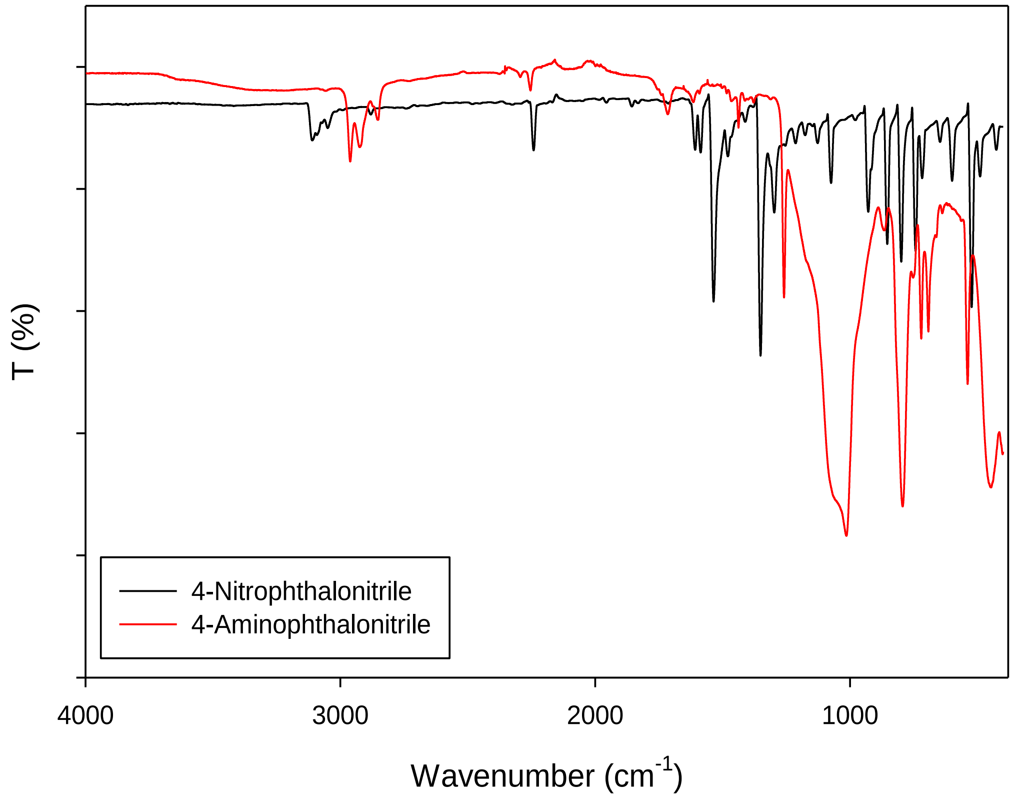

Figure S3: FT-IR spectrum of the products obtained upon the hydrolysis of TAPc and TNPc with sodium perrhenate ($NH_4Re_2O_4$) in the presence of triphenylphosphine (PPh_3).

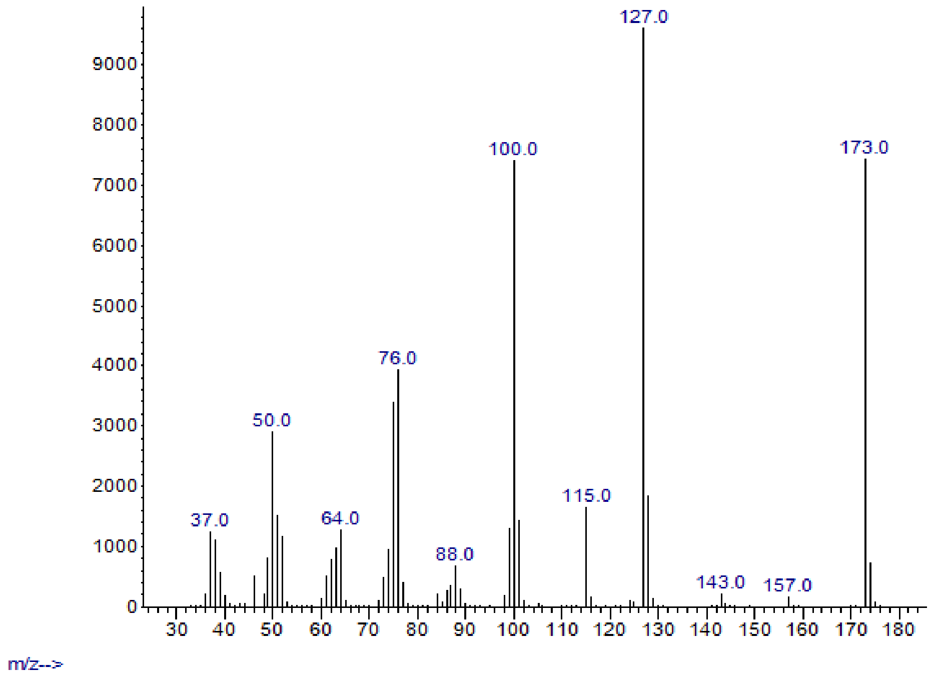

Figure S4: GC-MS spectrum of the clean product obtained upon the hydrolysis of tetranitro-phthalocyanine (TNPc) with sodium perrhenate ($NH_4Re_2O_4$) in the presence of triphenylphosphine (PPh_3).

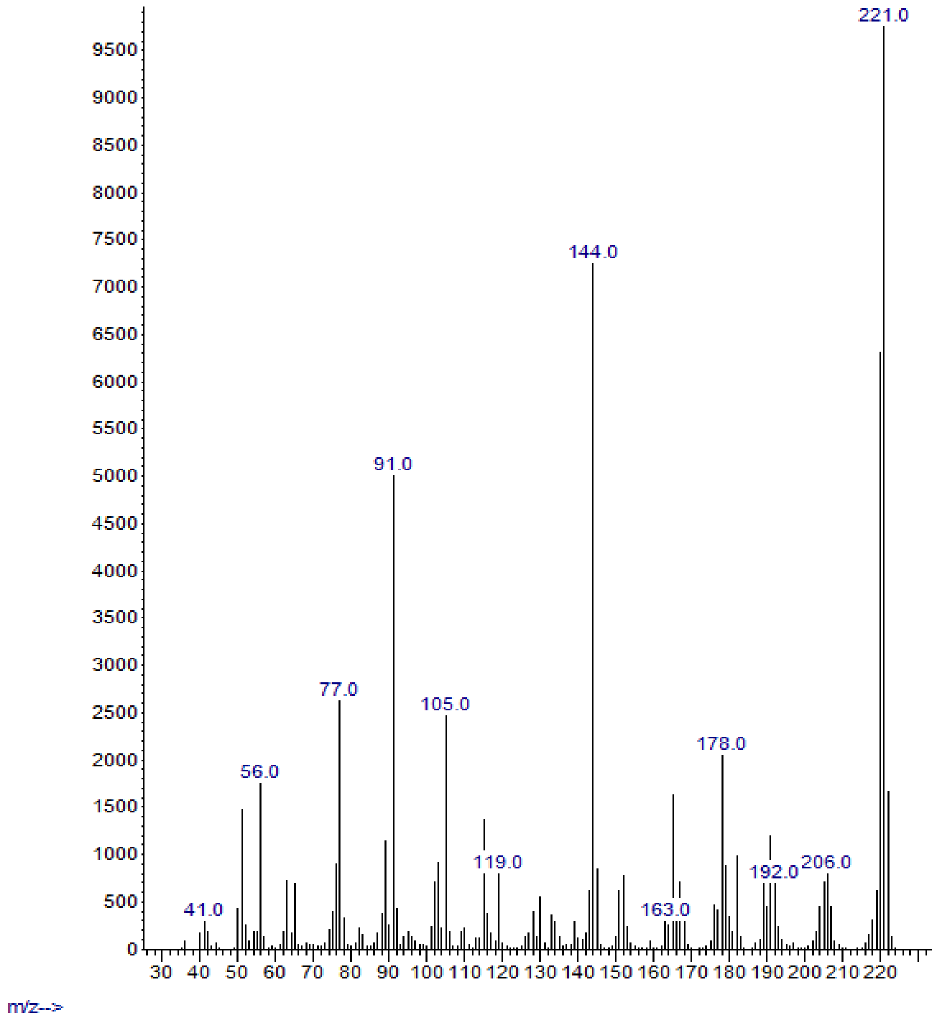

Figure S5: GC-MS spectrum of the crude products obtained upon the hydrolysis of tetraaminophthalocyanine (TAPc) with sodium perrhenate ($NH_4Re_2O_4$) in the presence of triphenylphosphine (PPh_3).

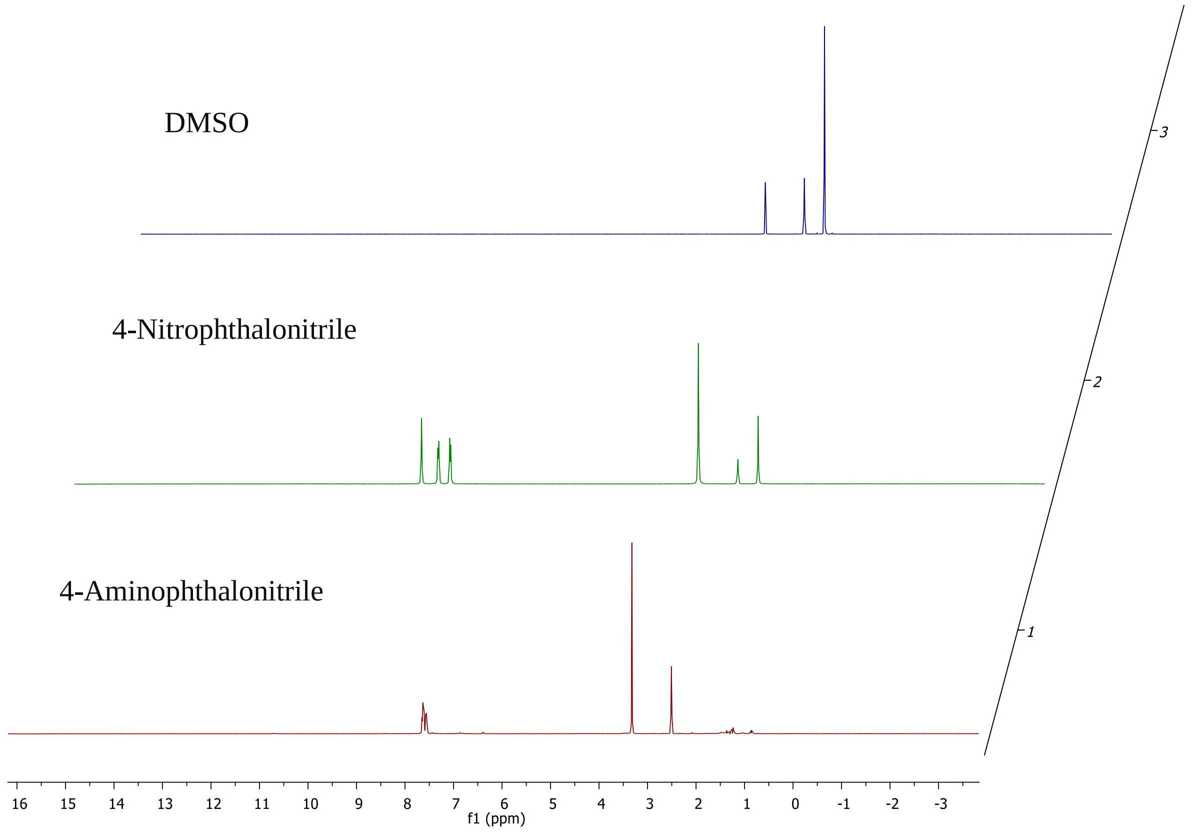

Figure S6: ^{1}H NMR spectrum of the hydrolysis products of TAPc and TNPc.

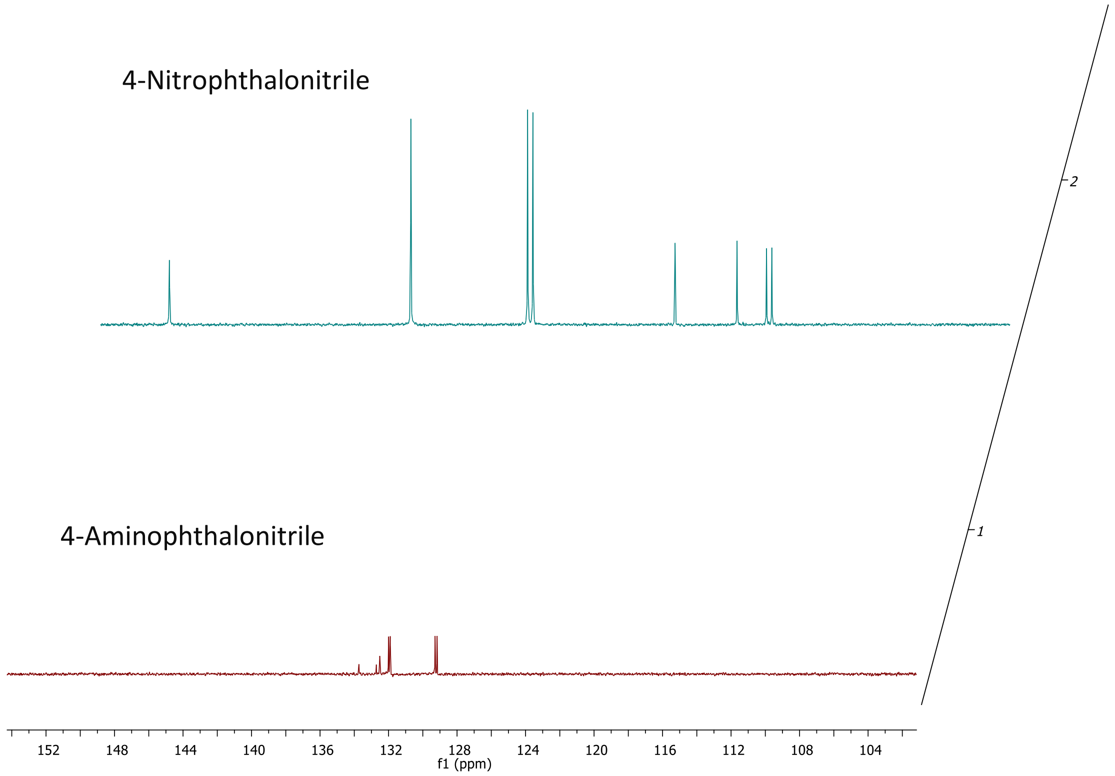

Figure S7: ^{13}C NMR spectrum of the hydrolysis products of TAPc and TNPc.

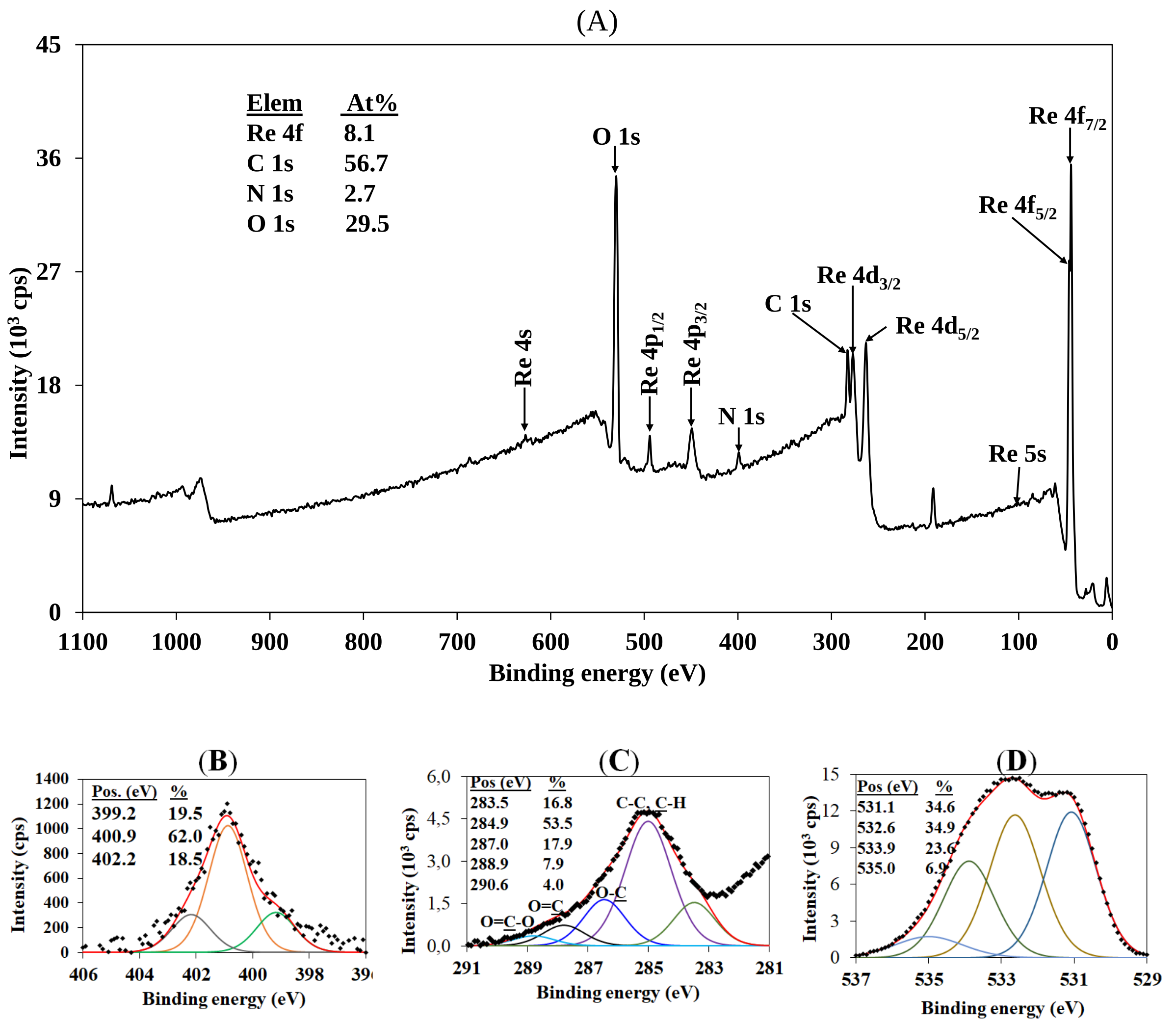

Figure S8: The XPS scans of Re_xO_y-TAPc-FA NPs (50 nm) (A) The wide scan, (B) high-resolution N 1s, (C) high resolution C 1s XPS spectra, and (D) high resolution O 1s XPS spectra

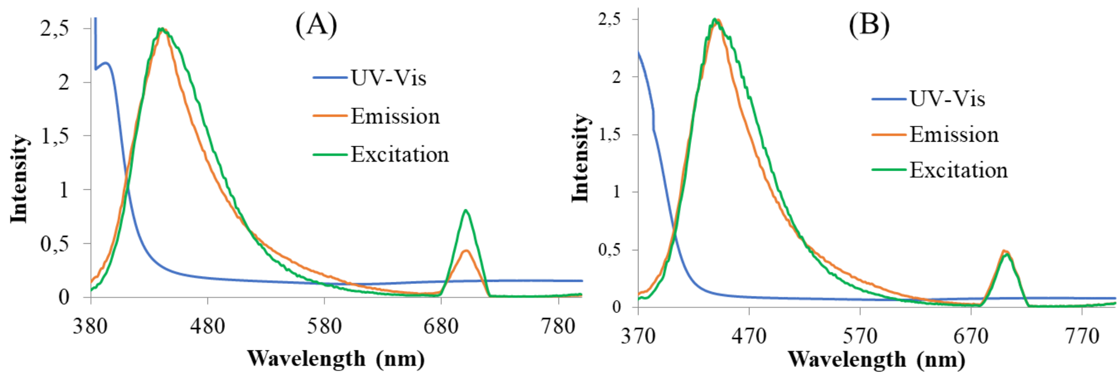

Figure S9: UV-Vis, excitation, and emission spectra of (A) 50 nm and (B) 10 nm Re_xO_y-TAPc-FA NPs

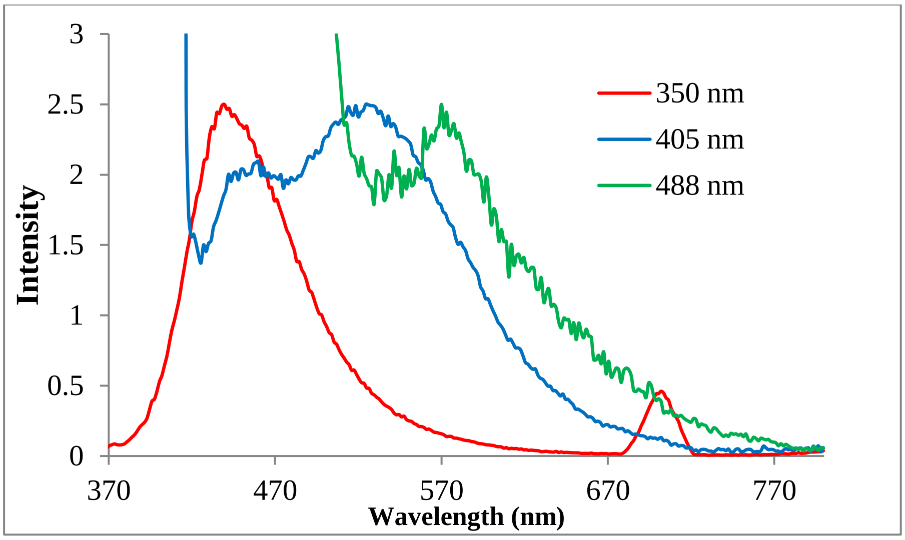

Figure S10: Emission spectra of size 10 nm Re_xO_y-TAPc-FA NPs excited at different wavelengths (350, 405 and 488 nm).

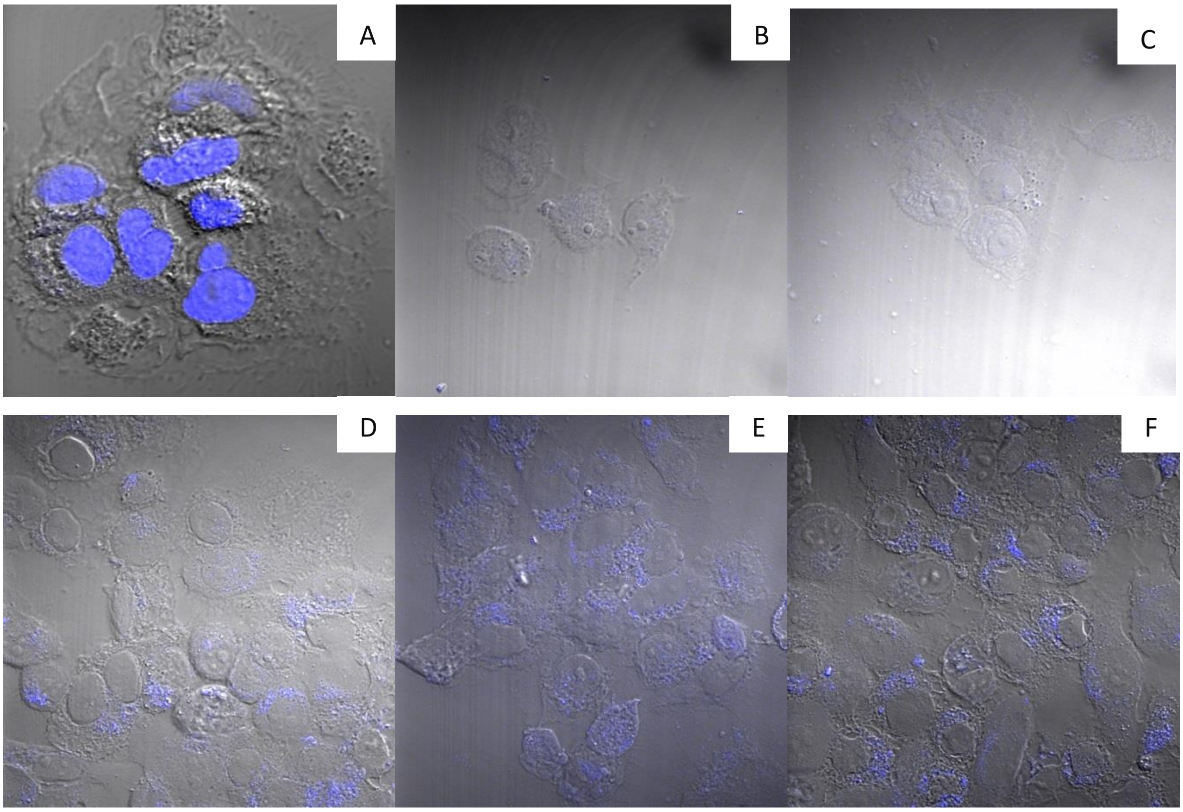

Figure S11: Confocal fluorescence images of HCC1806 cell lines treated with: (A) Hoechst 33324 nuclear stain, (B) Folic acid, (C) Methanol, (D) Uncapped Re NPs, (E) 50 nm and (F) 10 nm Re_xO_y-TAPc-FA NPs.

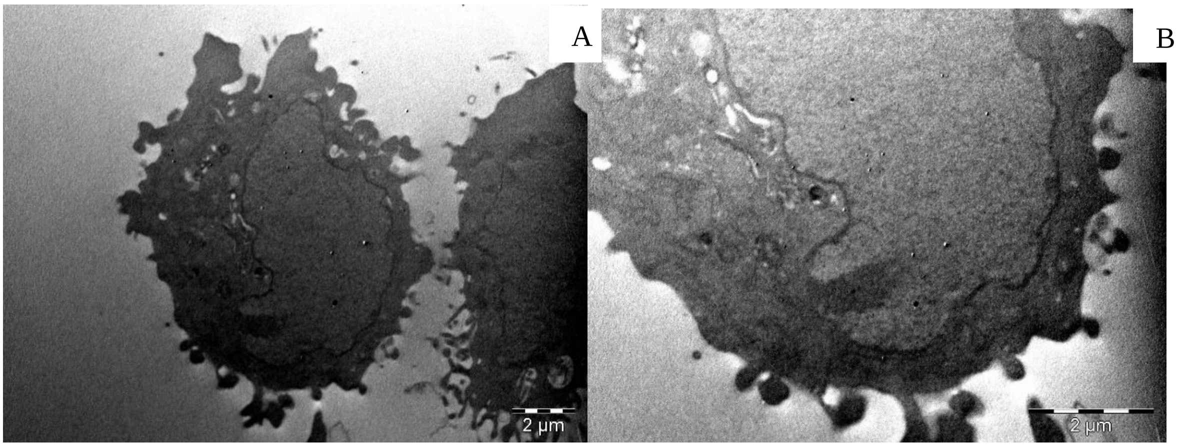

Figure S12: TEM images of cell sections treated with MeOH, (A) a full cell view and (B) a closer view (magnified). The scale bars indicate 2 µm in both images.

Table S1: Varied conditions used in the optimisation of Re_xO_y NPs synthesis.

**Sample code**	**Amount of NH_4ReO_4**	**Amount of FA-TAPc**	**Amount of $NaBH_4$**
SP1	60 µL	1000 µL	500 µL
SP2	80 µL	1000 µL	500 µL
SP3	60 µL	1500 µL	500 µL
SP4	80 µL	1500 µL	500 µL
SP5	60 µL	1000 µL	700 µL
SP6	80 µL	1000 µL	700 µL
SP7	60 µL	1500 µL	700 µL
SP8	80 µL	1500 µL	700 µL

Table S2: Summary of the absorption data of the Re_xO_y-TAPc-FA NPs and their corresponding average particle sizes.

**Sample code**	**SPR absorption (nm)**	**Average particle size (nm)**
SP1	391	22.85(0.04)
SP2	396	49.80(0.56)
SP3	—	—
SP4	383	21.94(0.58)
SP5	385	7.43(0.31)
SP6	392	18.66(0.05)
SP7	—	3.94(0.08)
SP8	380	8.63(0.92)

Table S3:Z-stack results obtained on 10 nm Re_xO_y-TAPc-FA NPs

**Depth (μm)**	**Phase contrast-fluorescence microscopic images**	**Normal cell view**
1	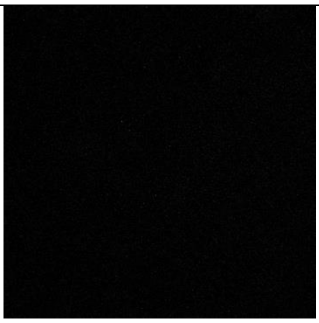	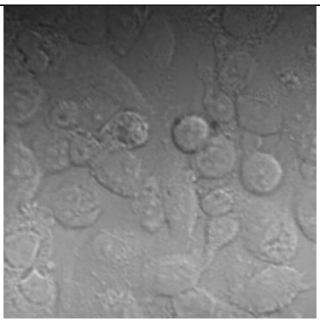
4	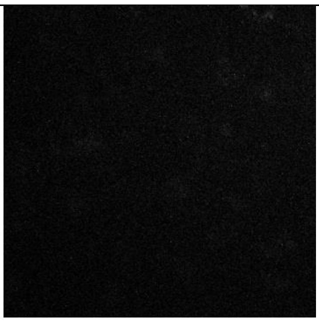	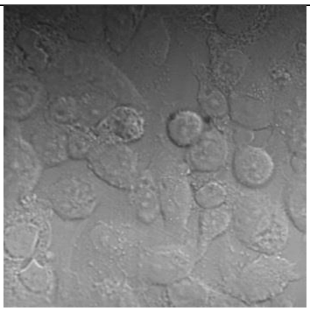

Table S3: Continued.

Depth (µm)	Phase contrast-fluorescence microscopic images	Normal cell view
8	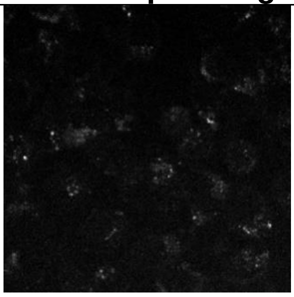	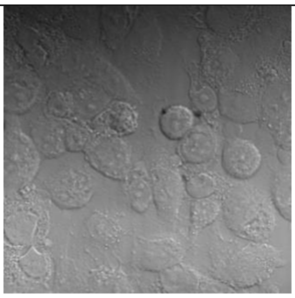
12	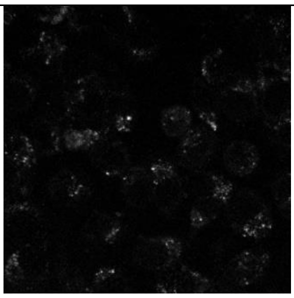	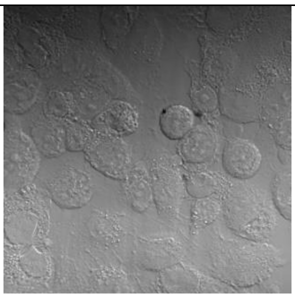
16	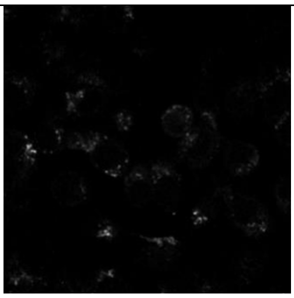	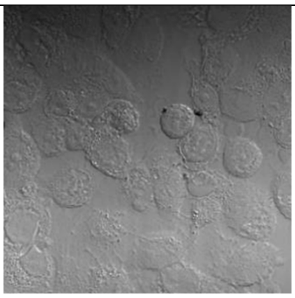
20	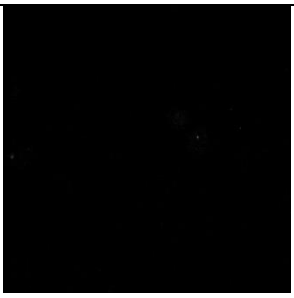	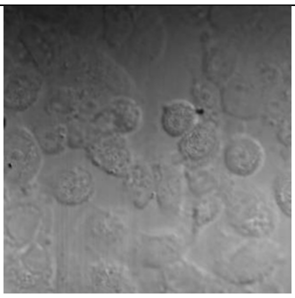
24	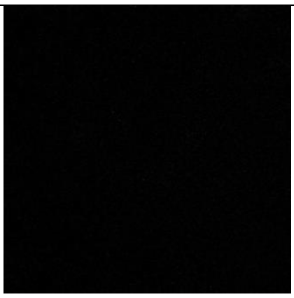	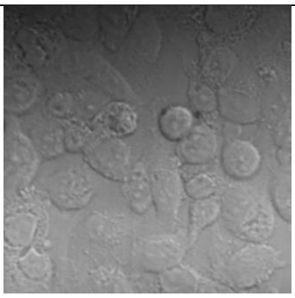

References

[1] C. D. Wagner, A. V Naumkin, A. Kraut-Vass, J. W. Allison, C. J. Powell, J. R. Rumble, **2003**, 911–919.

[2] J. Alzeer, P. J. C. Roth, N. W. Luedtke, *Chem. Commun.* **2009**, *15*, 1970–1971.

[3] R. O. Ogbodu, E. Antunes, T. Nyokong, *Polyhedron* **2013**, *60*, 59–67.

[4] J. A. De La Mare, J. C. Lawson, M. T. Chiwakata, D. R. Beukes, A. L. Edkins, G. L. Blatch, *Invest. New Drugs* **2012**, *30*, 2187–2200.
